# C-Terminal Residue of Ultrashort Peptides Impacts on Molecular Self-Assembly, Hydrogelation, and Interaction with Small-Molecule Drugs

**DOI:** 10.1038/s41598-018-35431-2

**Published:** 2018-11-20

**Authors:** Kiat Hwa Chan, Wei Hao Lee, Ming Ni, Yihua Loo, Charlotte A. E. Hauser

**Affiliations:** 1 Division of Science, Yale-NUS College, 16 College Avenue West, Singapore, 138527 Singapore; 2 Department of Chemistry, Krieger School of Arts & Sciences, 3400 North Charles Street, Johns Hopkins University, Baltimore, Maryland USA; 3 School of Biological Sciences & Engineering, Yachay Tech University, Hacienda San José s/n, San Miguel de Urcuquí, 100105 Ecuador; 4 Institute of Bioengineering and Nanotechnology, 31 Biopolis Way, Singapore, 138669 Singapore; 5 Laboratory for Nanomedicine, King Abdullah University of Science and Technology, Thuwal, 23955-6900 Saudi Arabia

## Abstract

Single molecular changes on a tripeptide can have dramatic effects on their self-assembly and hydrogelation. Herein, we explore C-terminal residue variation on two consistent ultrashort peptide backbones, i.e. acetylated-Leu-Ile-Val-Ala-Gly-Xaa and acetylated-Ile-Val-Xaa (Xaa = His, Arg, Asn). The objective of this study is to identify candidates that can form hydrogels for small-molecule drug (SMD) delivery. Haemolysis and cytotoxicity (with human adipose-derived mesenchymal stem cells) assays showed that the new soluble peptides (Xaa = His, Arg) are cytocompatible. Gelation studies showed that all but acetylated-Ile-Val-Arg could gel under physiological conditions. Longer peptidic backbones drive self-assembly more effectively as reflected in field emission scanning electron microscopy (FESEM) and circular dichroism spectroscopy studies. Rheological studies revealed that the resultant hydrogels have varying stiffness and yield stress, depending on the backbone and C-terminal residue. Visible spectroscopy-based elution studies with SMDs (naltrexone, methotrexate, doxorubicin) showed that besides the C-terminal residue, the shape of the SMD also determines the rate and extent of SMD elution. Based on the elution assays, infrared spectroscopy, and FESEM, we propose models for the peptide fibril-SMD interaction. Our findings highlight the importance of matching the molecular properties of the self-assembling peptide and SMD in order to achieve the desired SMD release profile.

## Introduction

Self-assembling peptides are currently being actively explored for a variety of nanobiotechnological applications. Their popularity as molecular building blocks can be attributed to the ease of tunability of their properties: by adjusting either the length of the peptide sequence or the identity of the amino acid residue (of which there are 21 readily available natural amino acids to choose from), it is theoretically possible to generate a peptide for any desired application. Anti-amyloidogenic peptides (e.g. Lys-Leu-Val-Phe-Phe^1^, Leu-Pro-Phe-Phe-Asp^2^, and others^3,4^) have been designed for the inhibition of amyloidogenesis that is the hallmark of several debilitating diseases such as Alzheimer’s disease^5^, Parkinson’s disease^5^, and even atherosclerosis^6^. However, in addition to the primary structure of the peptide, higher-order structures that arise from self-assembly can contribute yet more possibilities for complex and functionally demanding applications. In this regard, bountiful applications, including, but not limited to, oil spill remediation^7–9^, optoelectronic biomaterials^10^, organic ferroelectrics^11^, bioelectronics^12^, stabilisation of functional membrane proteins^13^, and even bone tissue regeneration^14–17^ have become possible with mere peptide building blocks.

Various higher order structures have been observed before, including nanotapes, nanotubes, nanoparticles, and nanofibrils^18–21^. Among these nanostructures, nanofibrils play a prominent role in many biomedical applications^22–24^. In particular, many of these nanofibrils are capable of entrapping water to form hydrogels^25^. This is the basis for many tissue engineering applications, in which the peptide nanofibrils form the scaffold that the cells of interest grow on. In the ideal scenario, after the scaffold has supported the growth of the new tissue, the peptide nanofibrils would disassemble and be subsumed or degraded by the body, thus allowing the new tissue to be free of any foreign material^26^. For this purpose, many different peptide systems with various modifications to support the growth of different tissue types have been developed. In order to assemble materials with predictable properties, much effort has been made towards the elucidation of rules governing hydrogelation^27–29^ and the types of secondary or higher order structures formed upon peptide self-assembly^30,31^. For instance, Ryadnov *et al*. have proposed a modular approach towards fine-tuning the structure and properties of *α*-helical assemblies with their dual-heptapeptide system^32^. The varying fibril thickness and morphology of peptide structures resulting from the combinations of the two heptapeptide building blocks offer deep insight into how resultant structures rely heavily on peptide sequence.

The tunability and biocompatibility of short self-assembling peptides have been exploited for developing next-generation scaffolds for tissue engineering and therapeutics delivery. These designed peptides support the growth of cells and are capable of releasing bioactive factors in a controlled manner to promote cell proliferation and differentiation^33,34^. Much work has been done to understand how various factors can be adjusted to control the release of various factors from hydrogels. Koutsopoulos *et al*. have reported that the rate of release of various proteins from the Arg-Ala-Asp-Ala (RADA) peptide hydrogel is dependent on protein properties (e.g. molecular mass, isoelectric point)^35^; Branco *et al*. have made similar observations with the MAX8 peptide hydrogel^36^. Ultrashort peptide hydrogels^37,38^ have also been demonstrated to be able to deliver a drug payload, i.e. the anticancer drug oxaliplatin^39^ and antibacterial silver nanoparticles^40^. Oxaliplatin was conjugated to the self-assembling peptide, acetylated Leu-Ile-Val-Ala-Gly-Lys-NH_2_; as the oxaliplatin-peptide conjugate could also participate in self-assembly with the unconjugated peptides, this helped to regulate the release of oxaliplatin. On the other hand, the silver nanoparticles were released directly from the aqueous medium of the hydrogel.

In our previous work, we demonstrated that the self-assembly and hydrogelation of tripeptides is intimately sensitive to the molecular structure of the peptide^41^. Herein, we extend this idea to ask if a consistent hydrophobic backbone may buffer against such changes in macromolecular properties. In addition, we probed the effect of changing peptide molecular properties on its interaction with a small-molecule drug (SMD) and influence the release of the SMD. To this end, we utilised two different backbones i.e. acetylated Leu-Ile-Val-Ala-Gly and acetylated Ile-Val with different C-terminal residues (His, Arg, Asn). We assessed the cytotoxicities, rheological properties, morphology, circular dichoism spectroscopic properties of the new self-assembling peptides, and their interaction with three SMDs. The hexapeptides were indeed found to be less sensitive to changes in the C-terminal residues than the tripeptides. Nonetheless, changes were still observed, especially with regards to interaction of the peptide fibrils with SMDs. Intriguingly, besides electrostatic interaction, it was found that another factor, which is likely to be the molecular shape of the SMD, together with the C-terminal residue, plays an important role in peptide fibril-interaction. This has implications in the design of peptide hydrogels for SMD release applications.

## Results and Discussion

### Peptide Preparation and Assessment of Cytotoxicity

**Figure 1:**
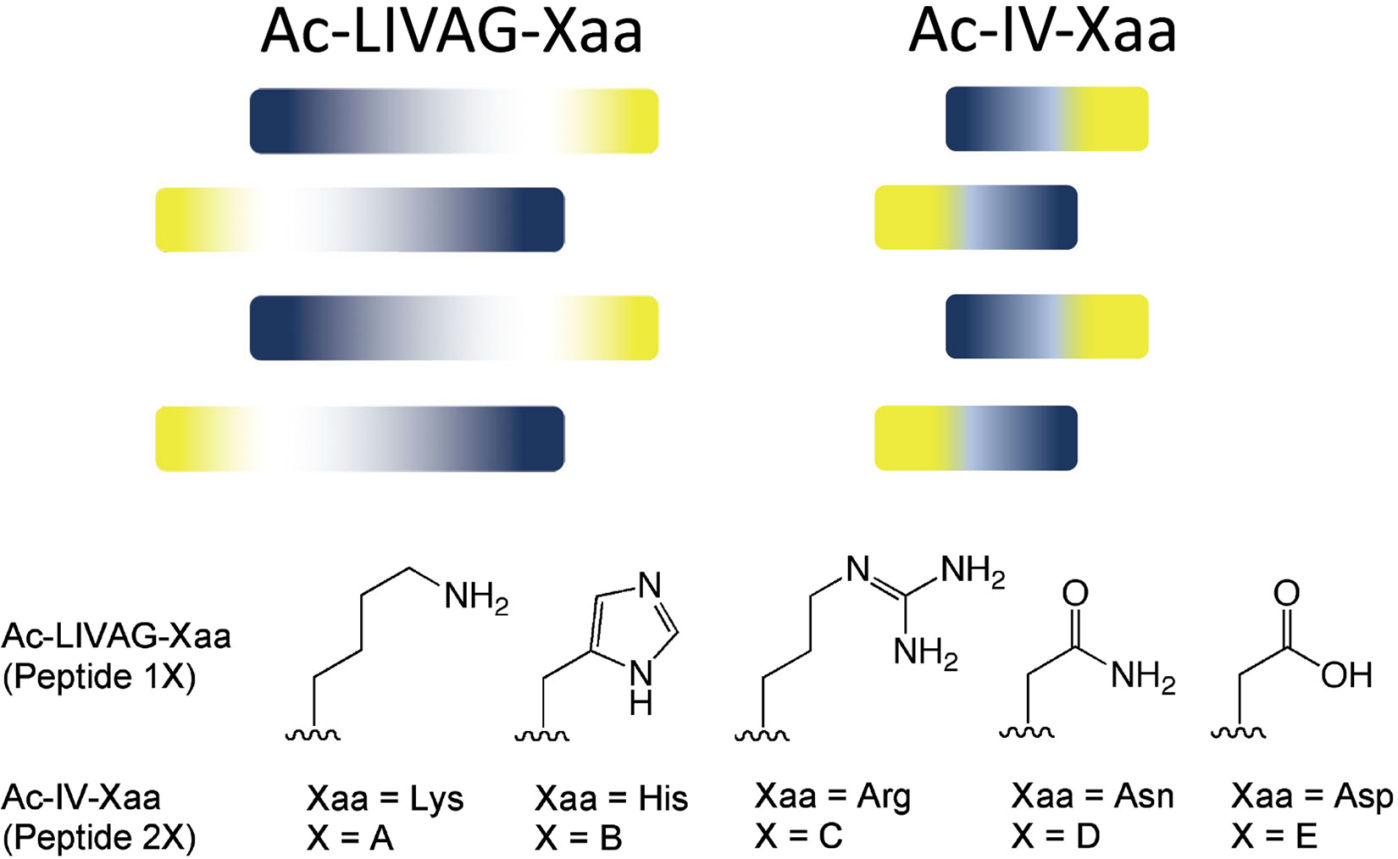
Depiction of how the hydrophobic amino acid backbones may self-assemble in an anti-parallel manner to accommodate the different sizes of the amino acid side chains. The darker the tone, the larger the side chain. Indigo refers to the hydrophobic amino acid and yellow refers to the hydrophilic C-terminal amino acid (Xaa). For X = (A–C) the peptides are amidated at the C-terminal (viz. a carboxamide); for X = (D, E) the C-terminal is a carboxylic acid.

The acetylated Leu-Ile-Val-Ala-Gly (Ac-LIVAG) and acetylated Ile-Val (Ac-IV) backbones have been previously reported to be nonpolar oligopeptidic backbones that promote peptide self-assembly in an antiparallel manner (Fig. 1)^37^. These studies have primarily focussed on peptides that have serine, threonine, cysteine, aspartic acid, glutamic acid, and lysine as the C-terminal amino acid residue. In this report, we expand the study to include histidine, arginine, and asparagine. Both histidine and arginine are basic amino acids, with histidine and arginine being less and more basic than lysine respectively. Asparagine possesses a neutral (carboxamide) side chain (under physiological conditions) and can be considered to be more basic than aspartic acid (carboxylic acid side chain). These new peptides (Fig. 1) will allow us to evaluate the effect of side chain basicity on self-assembly, hydrogelation, and peptide fibril interaction with small-molecule drugs (SMDs).

The peptides were prepared via standard solid-phase peptide synthesis. The purification of all peptides was straightforward, except for peptide 1D. Peptide 1D was found to be essentially insoluble in acetonitrile/water. As a result, it frequently precipitated on the reversed-phase column during HPLC (high performance liquid chromatography) purification with a gradient of acetonitrile/water, leading to poor yields (<10%). The solubilities of peptides 1 and 2 in phosphate-buffered saline (PBS; pH 7.4) to form clear solutions were then tested; peptides 1A and 2A were also assessed as references. The range of solubility for the peptides is shown in Table 1. Peptides 1D and 2D were only sparingly soluble in PBS. As Table 1 indicates, the hexapeptides dissolve to a lower extent than the tripeptides. This is reflective of the greater propensity of the hexapeptides to self-assemble and aggregate due to their longer hydrophobic backbone. In addition, peptides with lysine and arginine dissolve to a greater extent due to their charged side chains at pH 7.4.

**Table 1:** Soluble peptide concentrations and minimal peptide gelation concentrations in phosphate-buffered saline solution (pH 7.4).

Peptide	Soluble peptide concentration (mg/mL)	pK_a_	Zeta potential (mV)	Minimal peptide gelation concentration (mg/mL)	Status of gel
1A	3	~10.4	+33.40 ± 0.17	10	Clear, transparent
2A	10		+8.90 ± 1.05	15	Clear, transparent
1B	1	~6.8	+12.73 ± 1.27	25	Clear, translucent
2B	3		+8.47 ± 0.65	35	Clear, translucent
1C	3	~13.0	+17.90 ± 3.74	30	Opaque
2C	10		+9.53 ± 1.70	—	—

The pK_*a*_ values are approximations based on N-acetyl-L-lysinamide for 1A/2A, N-acetyl-L-histidinamide for 1B/2B, and N-acetyl-L-argininamide for 1C/2C, which in turn were calculated values based on the Advanced Chemistry Development (ACD/Labs) software v11.02. As the hydrophobic backbones are non-ionizable, they will only negligibly affect the pK_*a*_ of the C-terminal residue side chain. This will not affect the relative magnitudes of pK_*a*_ between peptides with different C-terminal residues. The zeta potential measurements were made in water at a peptide concentration of 10 mM at 25 °C. The values (mean of triplicate measurements) indicate that the fibrils formed by the hexapeptides are more positively charged than those of the tripeptides.

The cytocompatibility of the new peptides was assessed in order to ascertain their suitability for eventual drug delivery applications. The hemolytic ability was evaluated using rabbit red blood cells. This is a standard assessment of materials for biomedical purposes (protocol ISO 10993-4/NIH 77-1294)^42^, particularly for implants, hemostatics and wound dressings. Following red blood cell incubation with varying concentrations of peptide, the level of hemoglobin detected in the supernatant was comparable to the baseline values obtained in buffered solution. This indicates that the peptides do not disrupt the membranes of red blood cells, even at their respective maximal soluble concentrations. Since the peptides will not cause hemolysis, they can potentially be used as implantable scaffolds, drug delivery matrices and topical wound dressings.

**Figure 2:**
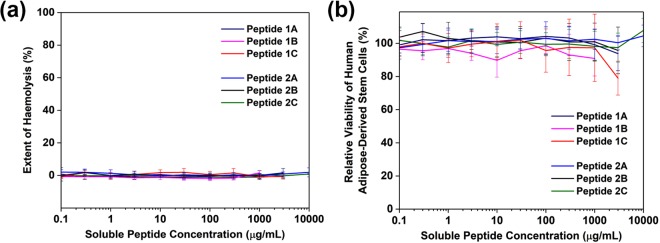
(**a**) Ultrashort peptides are non-hemolytic, even at their solubility limit. When mixed with rabbit red blood cells, the amount of haemoglobin released is comparable to the negative control, PBS. The detergent Triton X100 (1%) is used as the positive (lytic) control. The error bars represent the standard deviation of eight replicates. (**b**) The peptides are non-cytotoxic. Primary cells (hASCs) cultured in media containing solubilised peptides demonstrated comparable metabolic activity as untreated controls. The error bars represent the standard deviation of 24 replicates.

Human mesenchymal stem cells are multipotent stem cells that are being actively investigated for tissue engineering applications. They can be harvested from adipose tissue (hASCs) and are capable of differentiating into many tissue types, representing a very important source of stem cells for tissue regeneration. It is thus of interest to assess if the new peptide hydrogels are suitable as 3D matrices for the expansion and differentiation of hASCs^43–45^. A positive indication of cytocompatibility would be the survival of the cells following incubation with the peptides. For comparison, peptides 1A and 2A were included as part of the study. The cytocompatibilty of the peptides was evaluated by quantifying the metabolic activity using the WST-1 reagent and visualising the live/dead cells using calcein AM/ethidium homodimer staining. All the peptides demonstrated good viability, even at the solubility limit of the peptides (Fig. 2b). The metabolic activity was consistently comparable to control cultures (absence of peptides); the lone exception was peptide 2C with slightly lower (80%) metabolic activity. Fluorescence imaging of the hASCs stained with calcein AM and ethidium homodimer showed that the cells are metabolically active (presence of calcein fluorescence in the cells) and that the cellular membranes were intact (absence of ethidium bromide fluorescence in the cells); only peptide 1C demonstrated some ethidium bromide signal along with strong calcein fluorescence (Supplementary Fig. S1). This bodes well for the development of these peptides as implantable scaffolds.

We assessed the cytocompatibility with these two techniques as they offer differing insights to how amphiphilic peptides can interact with cells. As Lum *et al*. have found, although their cationic hydrophobic peptides were non-haemolytic, they exhibited substantial toxicity towards the two human cell lines that were used for assessment^46^. The balance between hydrophilicity and hydrophobicity is certainly very important in the design of self-assembling peptides, and this has certainly been achieved with the RADA (Arg-Ala-Asp-Ala) peptide^47^. Strikingly, this is a motif that is amenable to adjustment of the hydrophilic-hydrophobic components – when the hydrophilicity and hydrophobicity are respectively dialled down (changing Arg to Lys) and up (changing Ala to Leu), the resultant KLDL peptide continues to remain cytocompatible and biologically active^48^; similarly, when RADA was converted to EAKA (Glu-Ala-Lys-Ala), no difference in toxicity was measured^49^. This is a flexibility that we also observe with the Ac-LIVAG- and Ac-IV- consistent backbones: interconversion of the C-terminal residue among Lys, His, or Arg preserves the cytocompatibility of the peptides while introducing variability to the side chains.

### Rheological Studies of Peptide Hydrogels, Field Emission Scanning Electron Microscopy Studies of Peptide Nanostructures, and Circular Dichroism Spectroscopy Studies of Peptide Solutions

**Figure 3:**
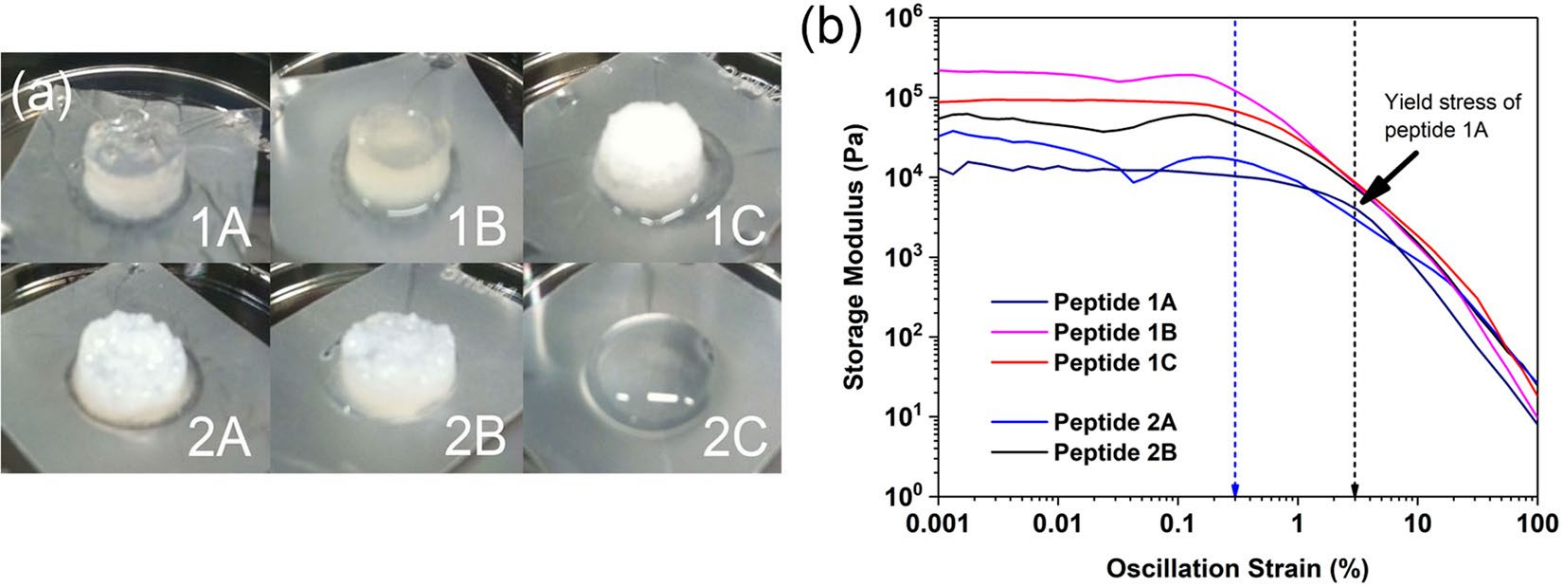
(**a**) Illustration of the hydrogels formed by peptides 1(A-C) and 2(A,B) at 40 mg/mL in phosphate buffered saline (PBS). Peptide 2C does not form a hydrogel in PBS even at 40 mg/mL. Only peptides 1A and 1B can form transparent hydrogels. (**b**) Rheological studies of the peptide hydrogels (40 mg/mL in PBS). The oscillation strain sweep studies were carried out at a constant angular frequency of 6.28 rad/s. The error bars, which represent the standard deviation of three separate measurements for each data point, have been removed for clarity. The black dotted line indicates the yield stress for peptide 1A hydrogel; the blue dotted line indicates the approximate yield stress for the other hydrogels. The graph illustrates that peptide 1A is unique among the peptide hydrogels in possessing a yield stress (3%) that is 10*×* as large as the yield stress of the other hydrogels (*∼*0.3%).

Compared to peptide 1A, which was previously reported to gel at a low concentration of 10 mg/mL (6 mM) in phosphate-buffered saline (PBS)^50,51^, peptide 2A was found to gel at 18 mM. Although peptide 1C could form a gel at 40 mg/mL (30 mM), the hydrogel formed was opaque and inhomogeneous; this is a big contrast to peptide 1A that forms a clear and transparent hydrogel (Fig. 3a). Peptides 1D and 2D are only sparingly soluble and do not form self-supported hydrogels; only dense clumps of precipitate were observed. This is a big contrast to peptides 1E and 2E, which were previously reported to form a self-supported hydrogel even at low peptide concentrations. On the other hand, perhaps unsurprisingly, the tripeptides generally require higher concentrations to gel. Peptide 2A was previously reported to require a higher peptide concentration to gel than peptide 1A^50^. This has also been observed for peptide 2B, in which 30 mM is required (c.f. 15 mM for peptide 2A). However, 2C did not gel even at the highest peptide concentration (90 mM) assessed, remaining as a solution in PBS. As with peptide 1D, peptide 2D could not gel and only precipitated. These observations suggest that self-assembly and hydrogelation require an intricate balance of the hydrophilic and hydrophobic elements in the peptide motif. When the hydrophobic characteristics predominate, the resulting peptides demonstrate low solubility and are more likely to form colloids or aggregate as precipitates in solution. When the hydrophilic components are dominant, the enhanced solubility reduces the driving forces for self-assembly. Consequently, hydrogelation is observed at markedly higher concentrations. Such a balance of hydrophilicity/hydrophobicity has been exploited (via enzymatic control) for the formulation of hydrogels^52,53^.

The stiffness of the peptide hydrogels was assessed via rheology. The peptides (peptides nX; n = 1–2, X =A-C) were dispersed in phosphate-buffered saline to final concentrations of 40 mg/mL. As Fig. 3a illustrates, only peptides 1A and 1B formed transparent hydrogels; the rest of the peptides (except for peptide 2C that did not gel) formed opaque hydrogels. This implies that the fibrils of peptides 1A and 1B had sufficient time to disperse in solution before entrapping water to gel whereas peptides 1C, 2A, and 2B gelled quickly upon fibril formation, leading to opaque hydrogels. These observations indicate that the C-terminal residue affected the kinetics of hydrogelation, viz. fibril-water interactions, in which the Arg of peptide 1C is capable of mediating stronger hydrogen bonding interactions between the fibrils and the surrounding water (compared to Lys of peptide 1A). Thus, this suggests that the greater number of hydrogen bond donors/acceptors on Arg (seven) compared to Lys (three) or His (two) is responsible for mediating greater fibril-water interaction for the hexapeptides. In the case of tripeptide 2C, however, the strong peptide 2C-water interaction overwhelms self-assembly conferred by the 2-residue backbone (as for peptides 2A and 2B). These observations point to the importance of the balance between inter-peptide interaction and peptide fibril-water interaction in determining whether hydrogelation occurs or not. This has been found to be important in starch gels^54^, and this was precisely the consideration made in the design of various peptide nanostructures^55^ and a vancomycin hydrogel^56^.

The rheological studies show that the hydrogels (except peptide 2C that did not gel) exhibit a range of stiffness from 5 kPa to 100 kPa. One might expect that a backbone with five amino acid residues (c.f. two amino acid residues) would be able to serve as a more effective anchor for the self-assembly process, leading to similar fibrillar nanostructures and consequently similar hydrogelation properties. However, this is not the case: the stiffness of the peptide hydrogels 1(A-C) varied between 7–100 kPa whereas that of peptides 2(A,B) varied between 5–20 kPa (Supplementary Fig. S2). The oscillatory strain sweep also bears out the same point. As Fig. 3b illustrates, despite their different consistent backbones, peptides 2A, 3A, 1B, and 2B break down at around the same strain (0.3%); the exception is that of peptide 1A, which broke down only at 3% strain, i.e. 10× that of the other hydrogels. Evidently, the C-terminal residue exerts a sufficiently significant effect that the stiffness and the yield stress of the hydrogels varied widely. This is intriguing as (1) peptide 1A would exhibit a different stress yield from the other two hexapeptides (similar to each other) and (2) the two hexapeptides (1B, 1C) and two tripeptides (1A, 1B) would exhibit the same yield stress. While various external macroscopic factors (e.g. temperature, pH) and peptide sequence have been reported to influence the rheological properties of hydrogels^57^, this is the first instance, to the best of our knowledge, that a molecular change in a single residue of the peptide or length of the hydrophobic backbone are found to impact upon these rheological properties so profoundly.

**Figure 4:**
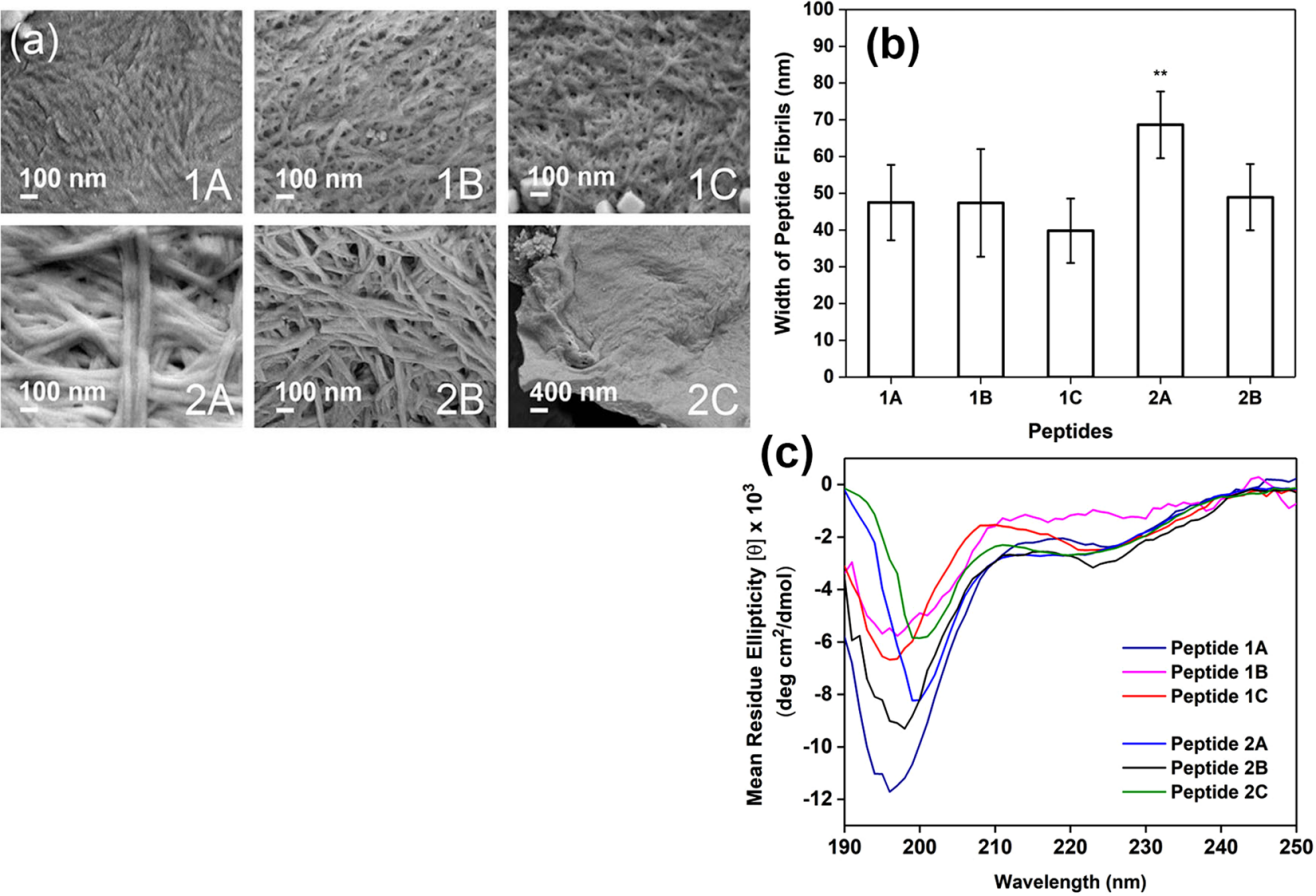
(**a**) Field emission scanning electron micrographs of the nanostructures within the various peptide hydrogels (20 mM in PBS). The electron micrographs were taken at 80,000 for peptides 1(A-C) and 2(A,B), and at 20,000× for 2C. Nanofibrils can be observed for peptides 1(A-C) and 2(A,B). (**b**) Tabulation of the mean fibril width for the various peptides. The error bars represent the standard deviation of 10 separate measurements. There is no significant difference among the mean fibril widths of Peptides 1(A-C) and 2B, which vary between 40–50 nm. **Only the mean fibril width of peptide 2A (mean = 68 nm, p < 0.05) was significantly different from the other peptides. (**c**) Circular dichroism spectra of the peptides (in PBS) at their respective soluble concentrations as reflected in Table 1. The CD spectra illustrate that the peptides possess polyproline II helical structures in solution, but the different intensities of the negative peaks (197 and 225 nm) suggest that the extent of such helical structures differ for each peptide.

Field emission scanning electron microscopy (FESEM) was utilised to analyse and contrast the nanostructures within the peptide hydrogels. As Fig. 4a illustrates, the fibrils of peptide 1A are more condensed together compared to the distinct fibrils observable for peptides 1(B,C) and 2(A,B). While some fibrillar feature is observed for peptide 2C, the fibrils appear to be very closely stuck to each other. Analysis of the electron micrographs reveals that mean peptide widths of peptides 1(A-C) and 2B are similar; only the mean fibril width of peptide 2A differs significantly from the other peptides (Fig. 4b). The observation that the fibrillar morphologies and mean peptide widths of peptides 1(A-C) are similar, but not among peptides 2(A-C), suggests that the five-amino acid consistent backbone (Ac-LIVAG) is long enough to facilitate similar self-assembly of homologous peptides (Ac-LIVAG-Xaa) with a lesser impact by the C-terminal residue (Xaa). On the other hand, the two-amino acid consistent backbone (Ac-IV) appears to be too short to direct similar self-assembly of homologous Ac-IV-Xaa peptides, with their C-terminal residue playing a much more significant role.

It is apparent that the identity of the C-terminal residue affects peptide self-assembly to differing extents depending on the length of the hydrophobic backbone. As the electron micrographs illustrate, the morphology of the hexapeptide fibrils are quite similar whereas those of the tripeptide fibrils are quite different from each other (Fig. 4). While the impact of the side chain of the C-terminal residue on peptide self-assembly has not been reported before, the impact of the chirality of the C-terminal residue is known. In the elegant work of Wang *et al*.^58^, it was shown that a change in the chirality of only the C-terminal residue of Ile-Ile-Ile-Lys did not impede self-assembly, but affected the “handedness” of the peptide fibrils. The impact of this single chirality change on hydrogelation, though, is not known from this work. However, the work of Marchesan *et al*. reveals that for Phe-Phe-Val, the chirality of the C-terminal residue (Val) has to be the same as the second residue (Phe) in order for hydrogelation to proceed^59^. In addition, they found that the two enantiomers, i.e. ^*D*^Phe-Phe-Val and Phe-^*D*^Phe-^*D*^Val, turned out to gel at different rates and resulted in hydrogels with different storage moduli, just as we have observed with our C-terminal changes. All these results point to the influential role that the C-terminal residue plays in peptide fibril-water interaction in hydrogelation.

Circular dichroism (CD) spectroscopy was also utilised to study the secondary structures that arise from the self-assembly of the peptides in solution. As Fig. 4c illustrates, all the peptides form polyproline II-type helical structures in solution, which is suggested by the presence of two negative peaks close to 197 nm and 225 nm as reported by Rucker and Creamer^60^ and Shi *et al*.^61^. Among the hexapeptides, the different intensities of the negative peaks indicate that the extent to which each hexapeptide forms the polyproline II helical structure in solution is different. This suggests that the various C-terminal residues of the hexapeptides have led to differences in peptide self-assembly. On the other hand, the shorter backbone of the tripeptides has led to even bigger effects on self-assembly: not only are the intensities of the negative peaks different, the peak wavelength is different for peptides 2A and 2C, which exhibit a red shift in one of the negative peaks from 197 nm (for peptide 2B) to ~200 nm, which suggests a significant difference in their polyproline II helical structure compared to the rest of the peptides. Thus, these CD studies also illustrate the profound impact both the backbone length and C-terminal residue exert on peptide self-assembly.

### Studying the Interaction of Peptide Fibrils and Small-Molecule Drugs via Elution Assay

**Figure 5:**
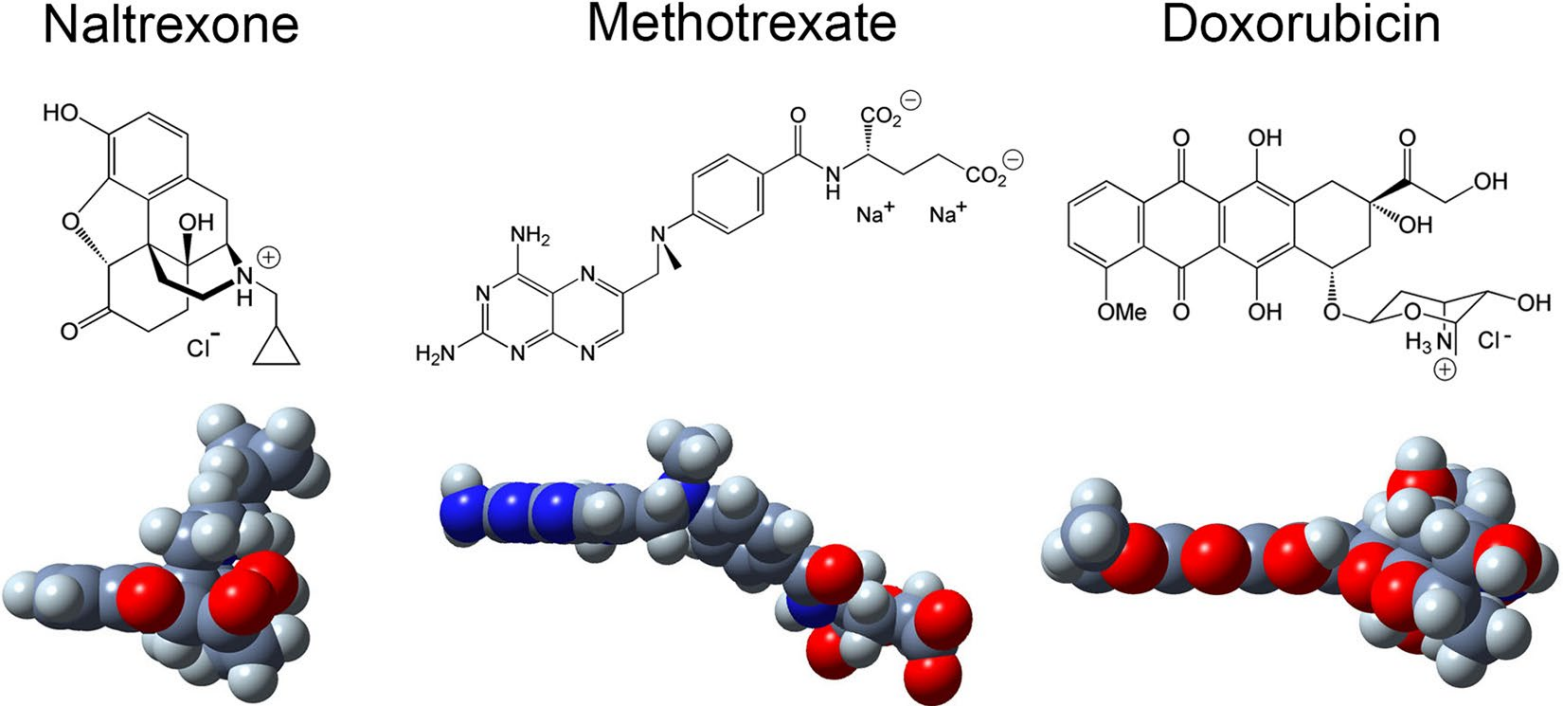
(Upper) Chemical structures of small-molecule drugs (SMDs), i.e. naltrexone, methotrexate, and doxorubicin. (Lower) Space-filling models of the SMDs: carbon (dark grey), hydrogen (light grey), nitrogen (blue), oxygen (red). Using an analogous procedure as Nagai *et al*.^89^, the structures of the SMDs were generated using Tinker (https://dasher.wustl.edu/tinker/) with a CHARMM19 force field and produced with GaussView.

Three medically relevant small-molecule drugs (SMDs), i.e. naltrexone, methotrexate, and doxorubicin, were used to study how the C-terminal residue affects the interaction between the peptide fibrils and SMDs. Naltrexone can be used to treat pruritus^62^, methotrexate can be used to treat psoriasis, arthritis, and even certain cancers^63^, and doxorubicin has been a prominent anticancer drug for decades^64^. Naltrexone, methotrexate, and doxorubicin were selected to study the effect of charge and molecular shape in their interaction with the peptide fibrils. At physiological pH 7.4, naltrexone (pK_*a*_~8.1)^65^ and doxorubicin (pK_*a*_~8.3)^66^ are positively charged while methotrexate is negatively charged (pK_*a*_~11)^67^. Molecular modelling shows that naltrexone is a relatively spherical molecule, methotrexate is a semi-planar molecule, and doxorubicin is largely a planar molecule (Fig. 5).

**Figure 6:**
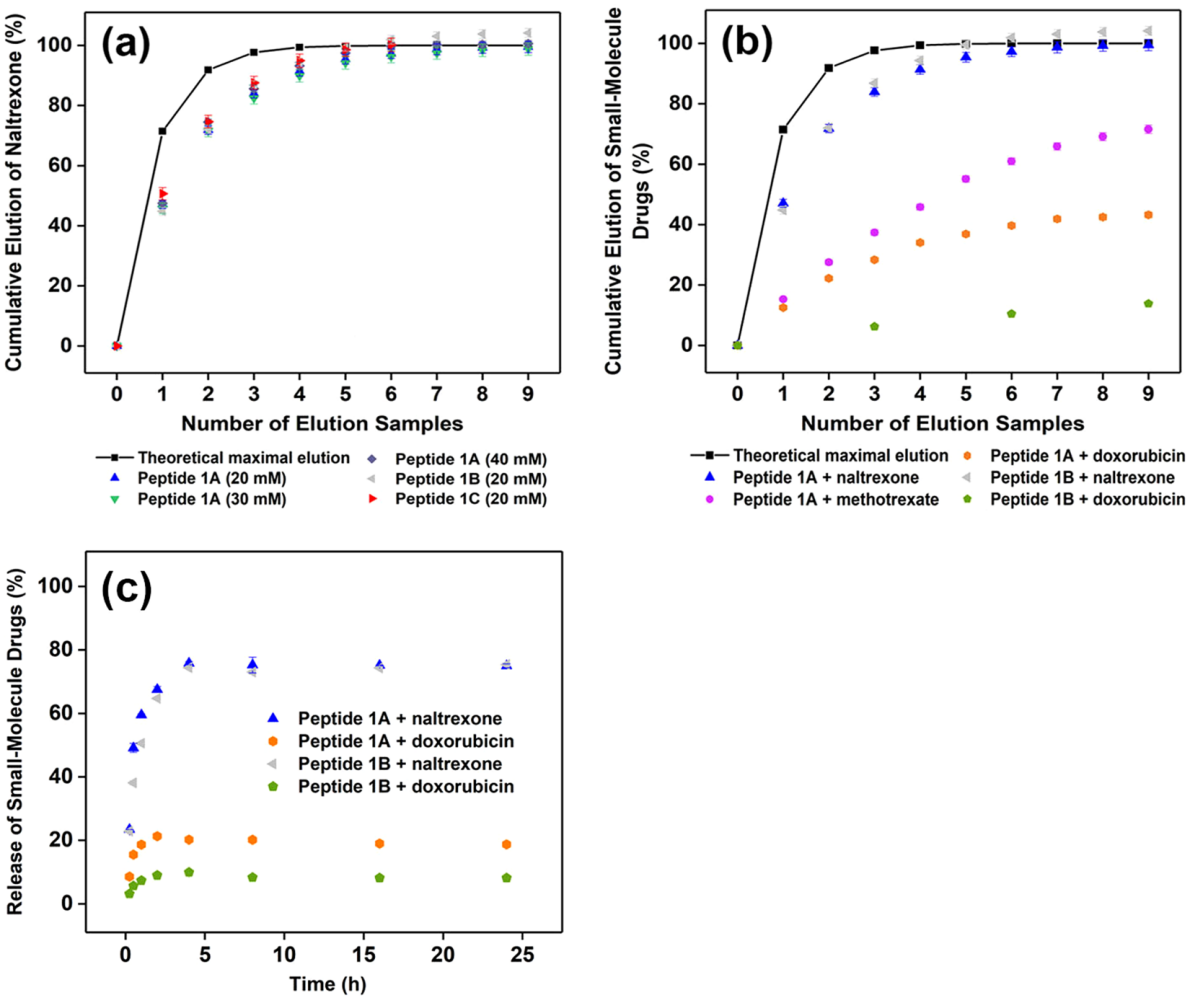
All the error bars represent the standard deviation of three separate measurements. Where error bars are not evident, they are smaller than the symbol. The solid line represents the theoretical maximal amount of SMD that would diffuse from the hydrogel into the extra volume of PBS if there were no obstruction to diffusion. (**a**) Elution of naltrexone (2 mM) from different concentrations of peptide 1A hydrogels (20–40 mM) and different peptide hydrogels (1A, 1B, 1C) at 25 °C. There is essentially no difference in naltrexone elution under these conditions. That the elution of naltrexone is less than the theoretical maximal elution for the first four samples does not necessarily imply there is interaction between naltrexone and the peptide fibril – a longer elution time before sampling will likely result in much a closer fit with theoretical maximal elution. (**b**) Elution of naltrexone (0.67 mg/g), methotrexate (0.89 mg/g), and doxorubicin (1.06 mg/g) from peptides 1A and 1B. The values in brackets correspond to the drug loading capacities of SMDs (2 mM) in the peptide hydrogels (20 mM). (**c**) Comparison of the time-dependent release of naltrexone and doxorubicin (2 mM each) from peptides 1A and 1B hydrogels (20 mM). The time points are 0.25 h, 0.50 h, 1 h, 2 h, 4 h, 8 h, 16 h, and 24 h. The graph shows that there was burst release of SMDs from the hydrogels, but the extent of release depends on the peptide-SMD combination. All the results indicate that the elution of SMDs depends both on the C-terminal residue (Xaa) of the peptide and the nature of the SMD.

In order to probe how well the SMDs interact with the positively-charged peptide fibrils (reflected by the zeta potential measurements in Table 1), we utilised a simple elution assay analogous to that employed by Branco *et al*.^68^: the SMD-encapsulated hydrogel, with a drug encapsulation efficiency of 100%, was layered with PBS (pH 7.4) for two hours, after which the PBS was removed and analysed for the amount of SMD eluted (Supplementary Fig. S3). Thus, the stronger the peptide fibril-SMD interaction, the lesser the amount of SMD that would be eluted. We compared the SMD elution profiles of the peptide hydrogels with the theoretical maximal amount of SMD that would diffuse from the hydrogel into the extra volume of PBS if there were no obstruction to diffusion. Figure 6a compares and contrasts the naltrexone elution profiles of different concentrations of peptide 1A hydrogels (20–40 mM) and (2) different peptide hydrogels (1A, 1B, 1C). It shows that the elution of naltrexone is essentially independent of peptide 1A concentration or peptide identity. One cause that could lead to such an effect is that there is minimal interaction of naltrexone with the peptide fibrils, viz. naltrexone is diffused within the water medium. This reason is also supported by the near-complete elution of naltrexone from the peptide hydrogels with sufficient elution counts (Fig. 6a).

While peptide hydrogels 1A, 1B, and 1C formed stable hydrogels with naltrexone, only peptide hydrogel 1A could do so with methotrexate (with some coagulation within the hydrogel); both 1B and 1C formed only hydrogel clumps with methotrexate and consequently could not be assessed with the elution assay. For doxorubicin, only peptides 1A and 1B formed stable hydrogels with it. Figure 6b illustrates the difference that the C-terminal residue and the SMD makes to the peptide fibril-SMD interaction. While the naltrexone elution profiles of peptide 1A and 1B are similar (blue and grey), their doxorubicin elution profiles are very different (orange and green): 40% of the encapsulated doxorubicin could be eluted from peptide 1A hydrogel whereas only 10% could be eluted from peptide 1B hydrogel. The time-dependent release of naltrexone and doxorubicin from both peptides 1A and 1B was also determined and compared with each other. As Fig. 6c illustrates, burst release of naltrexone and doxorubicin from both peptide hydrogels was observed. However, naltrexone was released to a greater extent than doxorubicin from both peptide hydrogels, which indicates the greater extent of interaction of doxorubicin (c.f. naltrexone) with the peptide fibrils. In addition, while naltrexone was released to a similar extent from either peptide 1A or 1B hydrogels, doxorubicin was released to a greater extent from peptide 1A (c.f. 1B), which reflects the greater interaction of doxorubicin with peptide 1B fibrils. These observations imply that the C-terminal residue (Xaa) plays an influential role in the interaction of the peptide fibril with different SMDs.

Given that both naltrexone and doxorubicin are positively charged at pH 7.4, their electrostatic interactions with peptide 1A (or peptide 1B) fibrils ought to be similar. However, since their elution profiles with each peptide hydrogel are different (1A: blue vs orange; 1B: grey vs green), this implies that another factor is at work. This factor is also evident by comparing the elution of different SMDs from peptide 1A hydrogels. The elution of methotrexate from peptide 1A hydrogel was lower (and slower) than that of naltrexone, and this might be attributable in part to the electrostatic attraction between the positively charged peptide 1A fibril and negatively charged methotrexate. However, despite being positively charged, doxorubicin was eluted to an even lower extent than the negatively charged methotrexate. Thus, there is a factor that helps the positively charged doxorubicin to interact with the positively charged peptide fibrils so as to reduce its elution.

**Figure 7:**
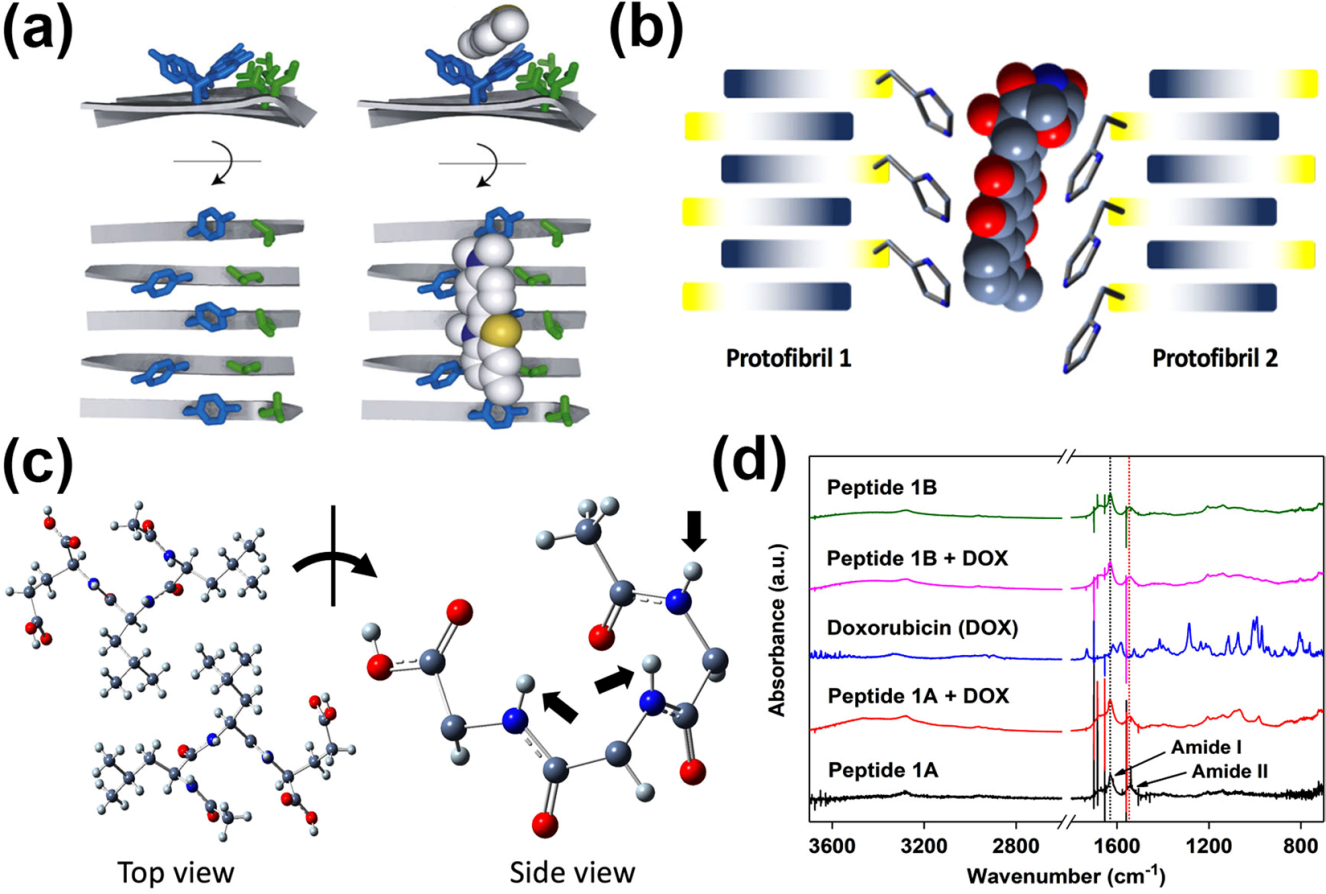
(**a**) Illustration of the Tyr and Leu ladders on the peptide self-assembly mimic (PSAM). The Tyr on five consecutive cross-beta strands of the PSAM binds thioflavin T via *π-π* interaction. This image is reprinted from J. Mol. Biol., 385, Biancalana, M.; Makabe, K.; Koide, A.; Koide, S., Molecular Mechanism of Thioflavin-T Binding to the Surface of *β*-Rich Peptide Self-Assemblies, 1052–1063, Copyright(2009), with permission from Elsevier^70^. (**b**) Proposed interaction of peptide 1B with doxorubicin. The imidazole side chains on two protofibrils of peptide 1B could together bind doxorubicin via *π*-*π* interaction. The structure of doxorubicin was generated and produced as outlined in the caption of Fig. 5. (**c**) Crystal structure of Ac-LLE^41^. This tripeptide packs in an antiparallel manner (top view), which leads to the amide protons (black block arrows) pointing towards the same face. The side chains of the tripeptide are omitted from the side view for clarity. The antiparallel arrangement of the hexapeptides could also align the amide protons on one face to interact with doxorubicin via amide-H mediated cation-*π* interaction. (**d**) Stacked plot of attenuated total reflection infrared spectra of various freeze-dried hydrogels. The plot shows that the presence of doxorubicin does not affect the *β* structures of the peptide fibrils, as indicated by the constant amide I absorption at 1635 cm^*−*1^.

As alluded to earlier, this factor could be the molecular shape of the SMD. Doxorubicin is a planar and (largely) aromatic molecule that could bind to the peptide fibrils in a manner analogous to thioflavin T, a planar aromatic molecular probe used in binding studies with peptide amyloid fibrils^69^. Due to the inherent insolubility and variability in binding of thioflavin T to peptide amyloid fibrils, the molecular details of the binding are currently still elusive. However, model studies utilising a peptide self-assembly mimic (PSAM) have shed light on this important interaction. Based on the molecular docking on an X-ray crystal structure of the PSAM^70^ and molecular dynamics simulation^71^, four possible binding sites were identified. The most prominent binding site comprises a hydrophobic groove that is bordered by two ladders (separated by one amino acid residue) along the long axis of the fibril: in one ladder, there are five Tyr side chains on five adjacent cross-beta strands; in the other ladder, there are five Leu side chains (Fig. 7a). Analogous to how acetylcholine esterase binds thioflavin T^72^, the PSAM is able to bind thioflavin T via a combination of *π*-*π* stacking (via Tyr side chains) and hydrophobic (via Leu side chains) interactions.

Since the Ac-LIVAG-Xaa class of peptides have been shown to assume cross-*β* peptide structures^37,50^, we propose that doxorubicin might bind analogously to the hexapeptides as thioflavin T binds to the PSAM. For peptide 1B, whose C-terminal residue is histidine, it has an aromatic imidazole side chain that could participate in *π*-*π* interaction^73^ with doxorubicin, just as how the aromatic phenolic side chain (of Tyr) in PSAM formed the “first ladder” that interacts with thioflavin T. (Fig. 7a) Although the imidazole side chains of the His residues on neighbouring antiparallel cross-*β* strands are five residues away on the same protofibril and are too far away from each other to form a binding site for doxorubicin via *π*-*π* interaction, the imidazole side chains can potentially form a binding groove with the side chains of the neighbouring protofibril instead (Fig. 7b). Intra-protofibril binding doxorubicin is possible, but this would have been mediated via imidazole NH*–π* interaction with doxorubicin (Supplementary Fig. S4). In fact, as Liao *et al*. noted^74^, the His NH– *π* interaction is stronger than *π*-*π* interaction, so it may be that both intra- and inter-protofibril binding of doxorubicin are relevant. Either mode of binding of doxorubicin should not affect the secondary structure of the peptide fibrils and this can be observed from the lack in change of the amide I absorption band (*ν* = 1635 cm^*−*1^), which is reflective of *β* structures^75,76^, in the presence of doxorubicin (Fig. 7d); electron micrographs also reveal little change in peptide morphology in the presence of doxorubicin (Fig. S5). As Lindberg *et al*. noted, the binding of thioflavin T by peptide fibrils of insulin also did not alter its structure^77^. Naturally, such binding interaction of doxorubicin with the peptide fibril would decrease its rate of elution from the hydrogel.

**Figure 8:**
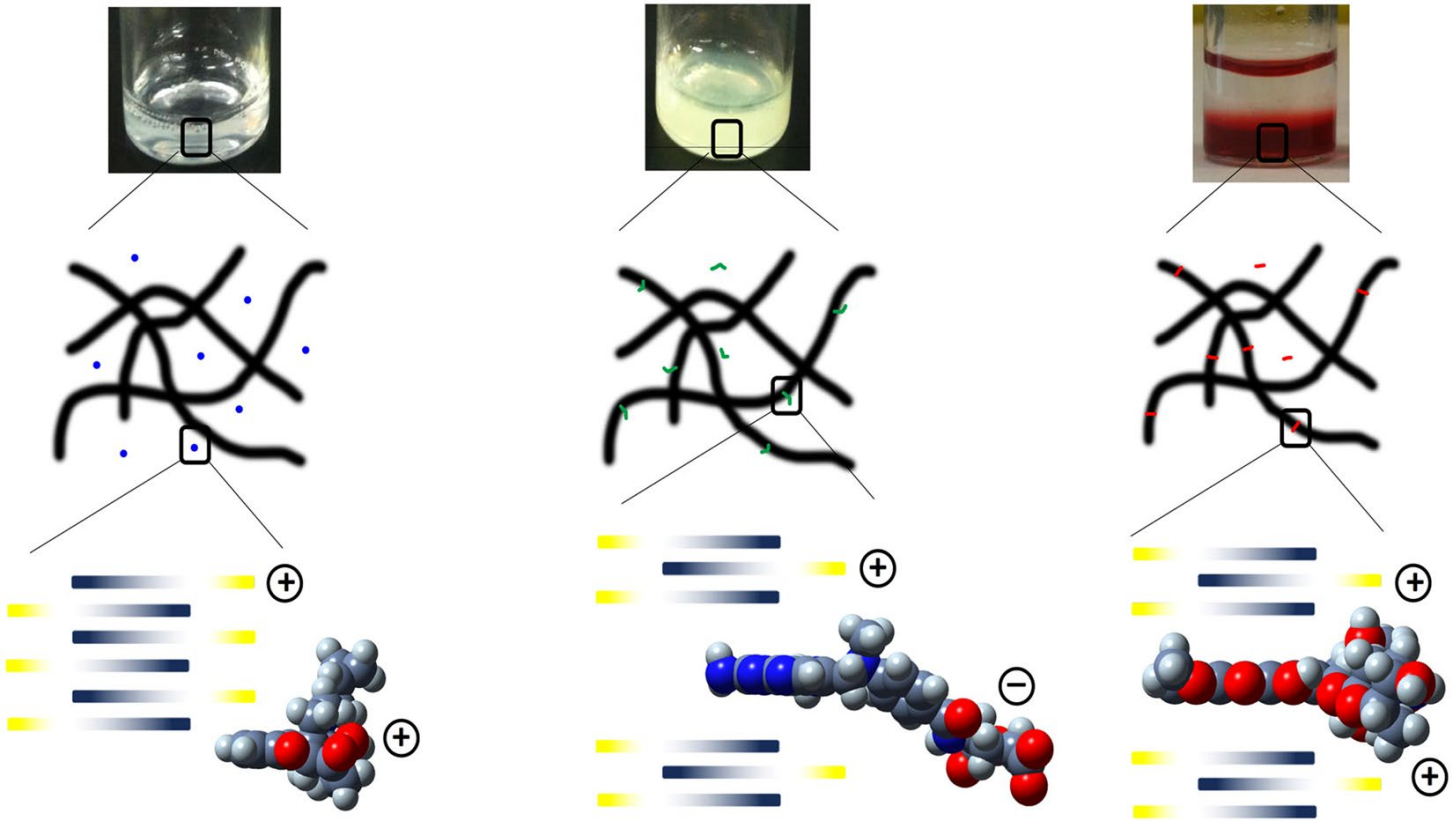
Illustration of the proposed interactions of the different drug release candidates with peptide hydrogels. (Left) Naltrexone is encapsulated within the hydrogel matrix with no interaction with the fibrils, (centre) methotrexate can interact electrostatically, but only intercalates partially with the peptide fibril, (right) doxorubicin can intercalate fully within the peptide fibril structure.

However, how would peptide 1A fibrils, which do not contain aromatic residues, interact with doxorubicin? As Wu *et al*.^71^ and Sabaté *et al*.^78^ noted, the positively charged thioflavin T binds poorly to PSAM and insulin fibrils respectively when there are positively charged residues close to the binding sites. Analogously, doxorubicin would bind poorly to peptide 1A fibrils which possess positively charged side chains. One possibility is that the hexapeptide may interact with doxorubicin via amide-H mediated cation-*π* interaction. It is known that cation-*π* interactions feature prominently in the stabilization of the tertiary structure of proteins^79^. In proteins, the cation-*π* interaction is commonly found to be mediated via the ammonium or guanidinium side chains of Lys and Arg with aromatic residues (Phe, Tyr, Trp)^80^. While the C-terminal residue of peptide 1A is Lys and could possibly interact with the aromatic surface of doxorubicin, the side chain of Lys is long (with four methylene units between the alpha carbon and the ammonium group) and projects the ammonium group into the aqueous medium of the hydrogel where the salt effect is strong and the corresponding cation-*π* interaction would be weak^81^. However, the amide proton can also participate in cation-*π* interactions, in which the partial positive charge on the amide proton is directed into *π* electron cloud of the aromatic ring^82–85^. As the crystal structure of Ac-LLE shows, the tripeptide packs in an antiparallel manner and this leads to the amide protons pointing in the same direction on one layer of Ac-LLE (Fig. 7c)^41^. Since the hexapeptide also self-assembles in an antiparallel manner, it is conceivable that the amide protons could be aligned in the same direction. Thus, the hexapeptides could interact with both faces of doxorubicin via amide-H mediated cation-*π* interactions, leading to the intercalation of doxorubicin into the peptide fibril at certain junctures (Fig. 8). Given that the doxorubicin:peptide ratio is 1:10, such intercalation should not affect the fibril structure significantly – as the infrared spectra and electron micrographs indicate, there is little change in the presence of doxorubicin (Fig. 7d, S5).

Naturally, if such cation-*π* interaction were to contribute to binding of doxorubicin to peptide 1A, then this binding mechanism would be operational for peptide 1B too. This could account for why doxorubicin was eluted to a lesser extent (and slower) from peptide 1B than from peptide 1A: there are two separate mechanisms binding doxorubicin to peptide 1B whereas there is only one mechanism for peptide 1A. Such cation-*π* interaction could also account for the greater (and faster elution) of methotrexate from peptide 1A hydrogel compared to doxorubicin. Methotrexate has a small planar component that could potentially intercalate into the fibril (Fig. 8) and it would understandably be less effective at doing so compared to doxorubicin given its smaller aromatic surface, leading to poorer binding. Given that the potentially small extent of intercalation and electrostatic attraction between the positively charged peptide fibril and negatively charged methotrexate still led to the greater (and faster) elution of methotrexate compared to doxorubicin, it implies that intercalation has a greater impact upon fibril-SMD interaction compared to electrostatic attraction. This is significant in view of the previous observation that negatively charged proteins were impeded in their release from hydrogels with positively charged peptide fibrils^36^. This would account for the lower (and slower) elution of doxorubicin relative to naltrexone even though both SMDs are positively charged at pH 7.4; presumably, the spherical shape of naltrexone would not enable it to intercalate within the peptide fibril (Fig. 8).

## Conclusion

In this study, we have found that a longer consistent hydrophobic backbone of acetylated Leu-Ile-Val-Ala-Gly can indeed serve as an anchor for self-assembly even as the C-terminal residue is varied. While the C-terminal does not affect the peptides’ cytocompatibility (non-haemolytic and non-cytotoxic), the stiffness and yield stress differ depending on the identity of the C-terminal residue. Thus, it might be possible to co-assemble different peptides (same backbone, different C-terminal residue) so as to produce hydrogels with a greater spectrum of tunability of various properties^86^. In addition, we have also found that the C-terminal residue plays an important role in affecting the rate and extent to which the peptide fibril interacts with an SMD. Interestingly, it might be that a planar aromatic SMD can interact with the peptide fibril via a novel intercalation mechanism. The details of this possible intercalation mechanism and the co-assembly of peptides are currently under investigation and will be reported in due course.

## Materials and Methods

All peptides were prepared via fluoren-9-ylmethoxycarbonyl (Fmoc)-based solid phase peptide synthesis and purified via reversed-phase high performance liquid chromatography-mass spectrometry (HPLC-MS) on an Agilent 6130 Quadrupole LC/MS system. Peptide hydrogels were prepared by weighing out the appropriate peptide mass in 2 mL-vials. After the requisite volume of phosphate-buffered saline (PBS) was added, the peptide-water mixture was shaken and sonicated for 10 s. The hydrogels were allowed to anneal for 24 h before they were used for further experiments. These procedures, along with the acquisition of precursors for peptide synthesis, were previously described in detail by us^41^. Naltrexone, methotrexate, and doxorubicin were purchased from Sigma-Aldrich. For the drug elution studies, a stock solution of 20 mM in Millipore water of each of these drugs was prepared. Methotrexate was neutralised with two equivalents of NaOH to produce the anionic form in solution. The stock solution was diluted to a final concentration of 2 mM in PBS prior to the preparation of the hydrogel for small-molecule drug elution studies (see below).

### Haemolysis assay of peptides with rabbit red blood cells

The procedure of Jiang *et al*. was followed^87^. Briefly, rabbit blood purchased from the Biological Resource Centre, Singapore, was centrifuged and the serum was removed. The red blood cells were rinsed with PBS, centrifuged, and the supernatant was removed; this process was repeated twice. The red blood cells were then incubated with the respective peptides in PBS in a 96-well plate (1 × 10^4^ cells/well) for 24 h at 37 °C. After that, the supernatant in each well was transferred with a multichannel pipettor into another 96-well plate and analyzed by a TECAN plate reader at 576 nm. 1% Triton-X100 was used as a positive (lytic) control. Each peptide was assessed for a total of eight times. The extent of haemolysis was calculated as: [(A_*peptide*_) − (A_*PBS*_)]/[(A_*Triton−X*100_) − (A_*PBS*_)] 100%, where A_*x*_ refers to the absorbance of the supernatant from the red blood cells that were treated with *x*.

### Cytotoxicity assay of peptides with hASCs

The hASCs were grown in DMEM culture medium, supplemented with 10% fetal bovine serum (FBS), at 37 °C, 5% CO_2_. Briefly, 1 × 10^4^ hASCs were seeded in each well and medium containing the peptide was added to the desired concentration. After incubation for 48 h at 37 °C, the medium was aspirated. Cell metabolic activity was measured with the Cell Proliferation Reagent WST-1 assay (Roche Diagnostics, Mannheim, Germany). Medium containing 10% WST-1 reagent was added to the cells. After 2 h, the supernatant was pipetted out into another 96-well plate, which was then analysed by a TECAN Infinite 200 plate reader at 440 nm (relative to the absorbance at 600 nm. Each peptide was assessed 24 times (3 × 8 samples per round) at each concentration. The positive control was a sample in which no peptide was added. The Live/Dead Viability/Cytotoxicity assay (Invitrogen, Carlsbad, CA, USA) was used to image the live and damaged cells. The samples incubated with peptide for 48 h were washed and incubated with 4 *μ*M of ethidium homodimer-1 and 2 *μ*M of calcein AM at room temperature for 20 min. After washing with PBS, fresh culture medium was added and the cells were imaged under a fluorescence microscope.

### Rheological studies of the hydrogels

Viscoelastic properties of the hydrogels were assessed at 25 °C with an ARES-G2 rheometer (TA Instruments, Piscataway, NJ) in the 25.0 mm-diameter titanium parallel plate geometry and a gap distance of 0.8 mm. For oscillatory frequency sweep studies, the plate rotation was varied between 0.1–100 rad/s at a constant strain of 0.1%. For oscillatory strain sweep studies, the strain was varied between 0.001–100% at a constant frequency of 6.28 rad/s. Each experiment was carried out in triplicates. This procedure was also previously described by us^88^.

### Field emission scanning electron microscopy (FESEM) study of peptide morphology

Peptide morphology was analysed with a JEOL JSM-7400F field emission scanning electron microscope. The freeze-dried sample was loaded onto the sample stage of the electron microscope and analysed under a vacuum of 10 Pa and a working current of 10A (5 kV). A detailed procedure was previously reported by us^41^. The diameter of the peptide nanofibers was determined by analysis of SEM images using ImageJ software (http://imagej.nih.gov/ij/docs/). Briefly, pictures were converted to 8-bit images and the threshold was adjusted in order to distinguish each nanofibril. Subsequently, the diameter of peptide nanofibrils was measured manually at 10 random locations in each micrograph.

### Circular dichroism spectroscopy studies of peptides

Circular dichroism spectroscopy studies were carried out with a Jasco J-1500 circular dichroism (CD) spectrometer, which was calibrated with (+)-camphor-10-sulfonic acid. A 0.1 mm quartz cuvette was utilised for the measurements, which was kept at a constant temperature of 20 °C with the Jasco CTU-100 circulating thermostat unit. The spectra were recorded at soluble peptide concentrations in PBS as reflected in Table 1 at a resolution of 1 nm.

### Zeta potential measurements of peptides

The zeta potential of the peptide fibrils was measured with an Anton Paar Litesizer 500 Particle Sizer. An omega zeta potential cuvette was utilised for the measurements, which was kept at a constant temperature of 25 °C. Triplicate measurements of each peptide sample were made at concentrations of 10 mM in water.

### Small-molecule drug elution studies with naltrexone, methotrexate, and doxorubicin

The peptides were weighed out in 4 mL-vials, after which phosphate-buffered saline (PBS) containing the small-molecule drug (SMD final concentration = 2 mM) was added to a final volume of 0.30 mL each. The peptide-SMD-PBS mixtures were then shaken and sonicated for 10 s, after which the mixtures were allowed to anneal for 24 h. After that, 0.75 mL of fresh PBS was slowly added with a pipettor to the surface of the hydrogel so as not to disturb the surface of the gel. After 24 h at 25 °C, the PBS was carefully extracted with a pipettor for UV/vis analysis; a fresh volume (0.75 mL) of PBS was then replaced. This process was repeated as many times as the number of pre-determined elution samples. The extracted PBS was transferred into a cuvette (pathlength = 10 mm) and analysed with an Agilent 8543 UV/visible spectrometer for the presence of the SMD. Naltrexone, methotrexate, and doxorubicin were respectively analysed at 280 nm, 390 nm, and 480 nm in PBS. A control experiment was set up for each peptide hydrogel, for which no drug was added. This is to account for absorption at the respective wavelengths due to eluted peptide. The elution profile of each peptide is compared with a theoretical maximal elution profile, viz. the theoretical maximal amount of SMD that would diffuse from the hydrogel into the extra volume of PBS if there were no obstruction to diffusion. The cumulative eluted percentage of SMD at each point is given by: [1 − (1/3.5)^*n*^] 100%, where *n* is the number of elution. The time-dependent release of naltrexone and doxorubicin was carried out in a similar manner to the elution studies, except that the supernatant was replaced on top of the hydrogel after each UV/visible spectroscopic measurement at each time point.

### Attenuated total reflection infrared spectroscopy studies of peptides

ATR-FTIR spectroscopy studies were carried out with a Perkin-Elmer Spectrum 100 IR spectrometer, which was fitted with a germanium-based PIKE MIRacle attenuated total reflectance (ATR) sampling accessory. Briefly, a freeze-dried sample of the hydrogel was loaded on the sample stage of the spectrometer. At least 16 scans were recorded. The spectra were recorded by subtracting a reference spectrum of air. A detailed procedure was previously described by us^88^.

## Electronic supplementary material

Supporting information
