# Supplementary material for: C-Terminal Residue of Ultrashort Peptides Impacts on Molecular Self-Assembly, Hydrogelation, and Interaction with Small-Molecule Drugs
Source: Sci Rep. 2018 Nov 20;8:17127. doi: 10.1038/s41598-018-35431-2 (PMC6244206; doi:10.1038/s41598-018-35431-2)
Supplement: Supplementary file 1 — Supporting information [file 41598_2018_35431_MOESM1_ESM.pdf]

Supporting Information for:

C-Terminal Residue of Ultrashort Peptides Impacts on Molecular Self-Assembly, Hydrogelation, and Interaction with Small-Molecule Drugs

Kiat Hwa Chan[1]*, Wei Hao Lee[2], Ming Ni[3], Yihua Loo[4], and Charlotte A. E. Hauser[5]**

[1] Division of Science, Yale-NUS College, 16 College Avenue West, Singapore 138527
[2] Department of Chemistry, Krieger School of Arts & Sciences, Johns Hopkins University, Baltimore, Maryland, USA
[3] School of Biological Sciences & Engineering, Yachay Tech University, Hacienda San José s/n, San Miguel de Urcuquí 100105, Ecuador
[4] Institute of Bioengineering and Nanotechnology, 31 Biopolis Way, Singapore 138669
[5] Laboratory for Nanomedicine, King Abdullah University of Science and Technology, Thuwal 23955-6900, Saudi Arabia

* kiathwa.chan@yale-nus.edu.sg
** charlotte.hauser@kaust.edu.sa

Contents 1

1. Live-Dead Assay of Peptides 1B, 1C, 2B, and 2C 2

2. Angular frequency sweep studies of the peptide hydrogels 3

3. Elution of small-molecule drugs (SMDs) from peptide hydrogels 3

4. Intra-protofibril binding of doxorubicin by peptide 1B 4

5. Field emission scanning electron microscopy studies of peptide hydrogel with SMDs 4

6. High performance liquid chromatography data of new peptides 5

7. Electrospray Ionisation mass spectrometry data of new peptides 7

1. Live-Dead assay of peptides 1B, 1C, 2B, and 2C

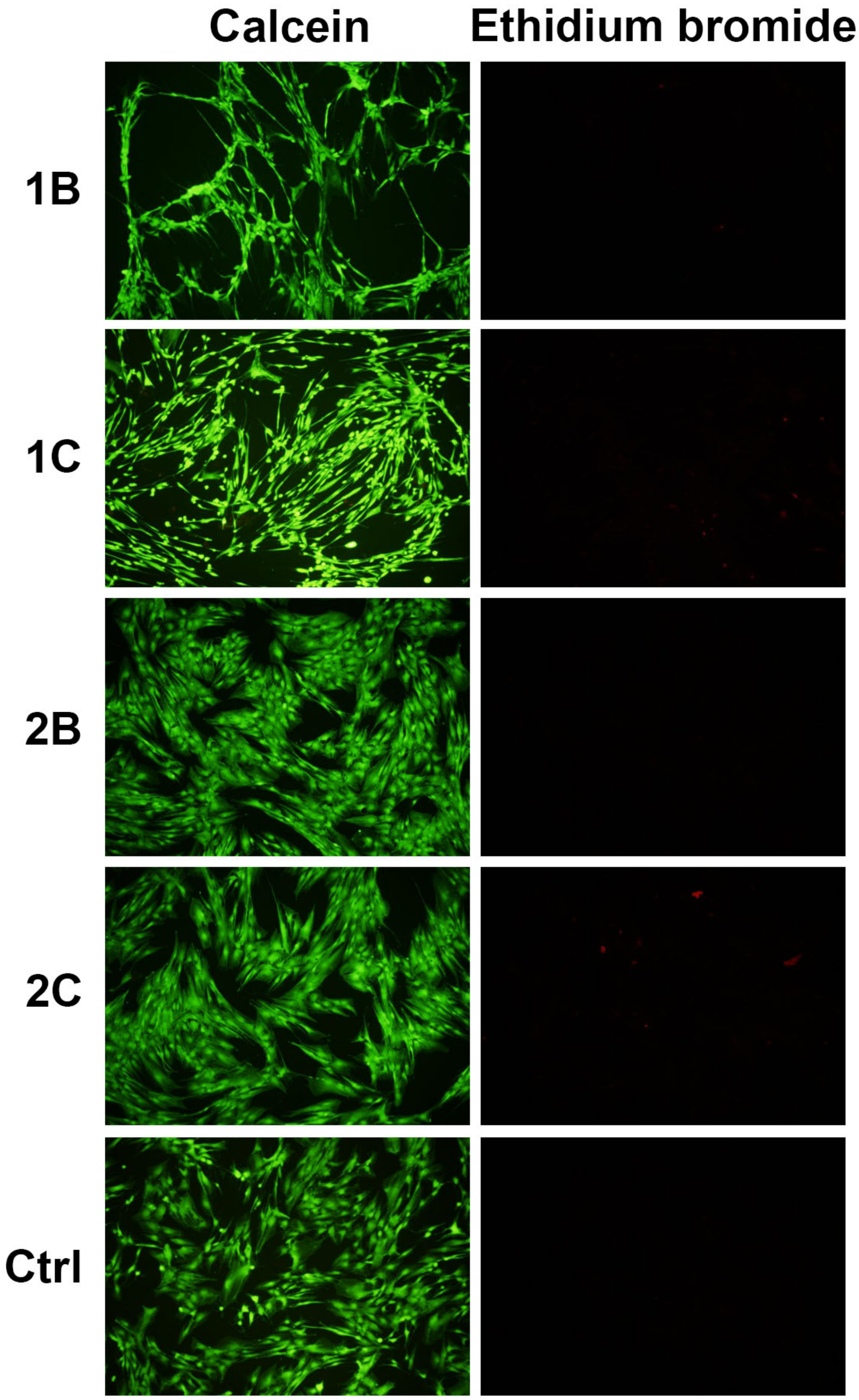

Figure S1. Calcein and ethidium bromide staining of human adipose-derived stem cells (hASCs) in the presence of peptides 1B, 1C, 2B, and 2C. The control refers to no addition of peptides. The calcein fluorescence images indicate that the presence of the peptides do not affect the metabolic activity of the hASCs while the ethidium bromide fluorescence images indicate that the peptides 1B, 1C, and 2B did not compromise the membrane integrity of the hASCs; only peptide 2C led to the entry of a very small amount of ethidium bromide (red fluorescence) into the hASCs.

2. Angular frequency sweep studies of the peptide hydrogels

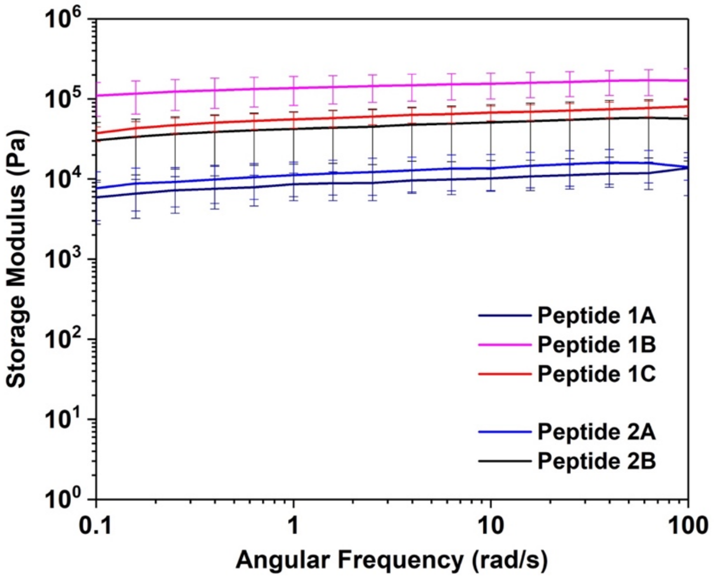

Figure S2. The rheological studies were carried out at a constant strain of 0.1 %. The error bars represent the standard deviation of three separate measurements. The graph shows that despite the consistent backbones, the stiffness of the hydrogels within each group can vary considerably: 7-100 kPa for the hexapeptides (1A, 1B, 1C) and 5-20 kPa for the tripeptides (2A, 2B). This shows that the C-terminal residues influence hydrogelation (i.e. interaction of the peptide fibrils with water) profoundly.

3. Elution of small-molecule drugs (SMDs) from peptide hydrogels

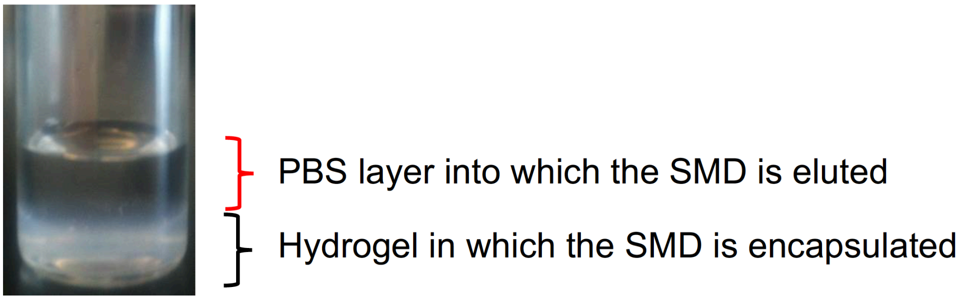

Figure S3. Experimental set-up to measure the rate and extent of elution of various SMDs from various peptide hydrogels.

4. Intra-protofibril binding of doxorubicin by peptide 1B

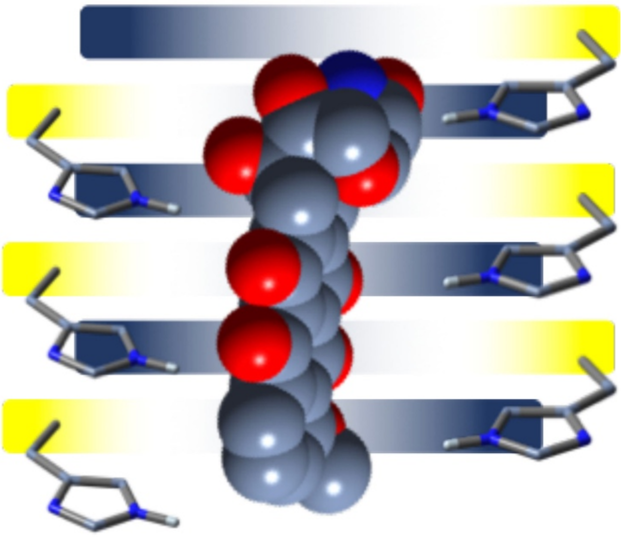

Figure S4. A proposed model of how intra-protofibril binding of doxorubicin by peptide 1B might occur. Except for the imidazole N-H, all other hydrogen atoms on the imidazole side chain of His are removed for clarity. The imidazole N-H can point towards the aromatic faces of doxorubicin and bind the SMD via N-H mediated cation-π interactions.

5. Field emission scanning electron microscopy studies of peptide hydrogel with SMDs

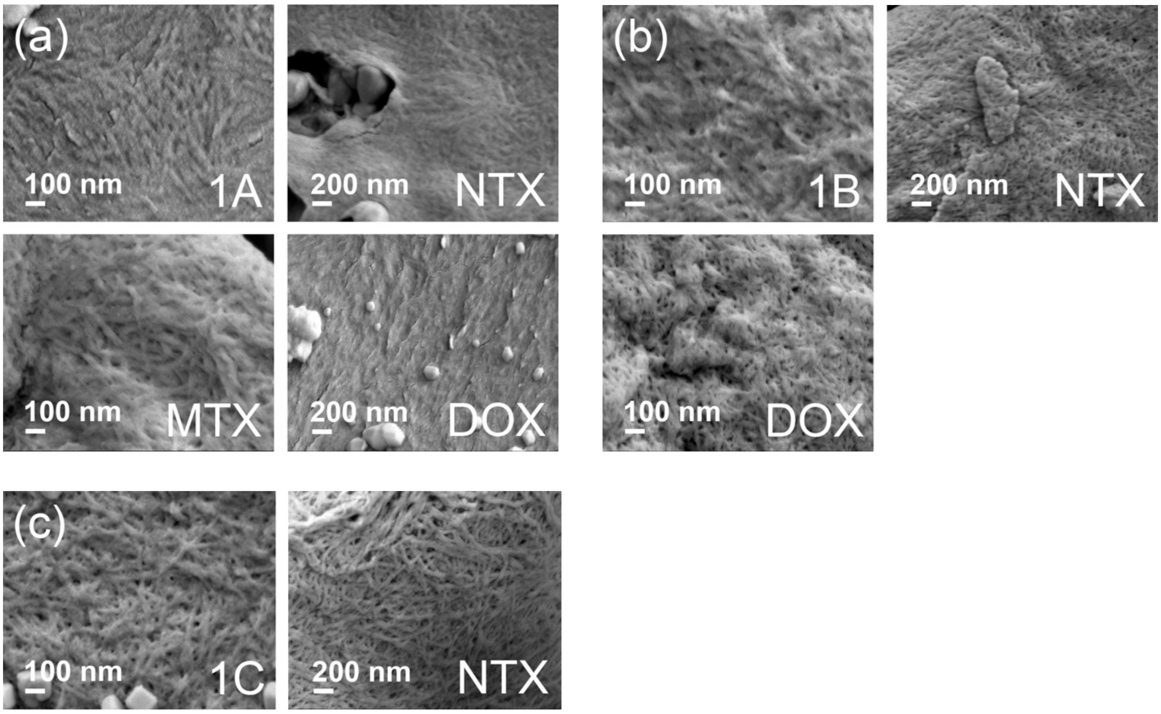

Figure S5. Scanning electron micrographs of various freeze-dried peptide hydrogels in the presence of various SMDs (10 molar %). (a) Peptide 1A hydrogels with naltrexone (NTX), methotrexate (MTX), and doxorubicin (DOX). (b) Peptide 1B with naltrexone (NTX) and doxorubicin (DOX). (c) Peptide 1C hydrogel with naltrexone (NTX). The electron micrographs show that the presence of the SMDs do not significantly affect the morphology of the peptide hydrogels.

6. High performance liquid chromatography data of new peptides

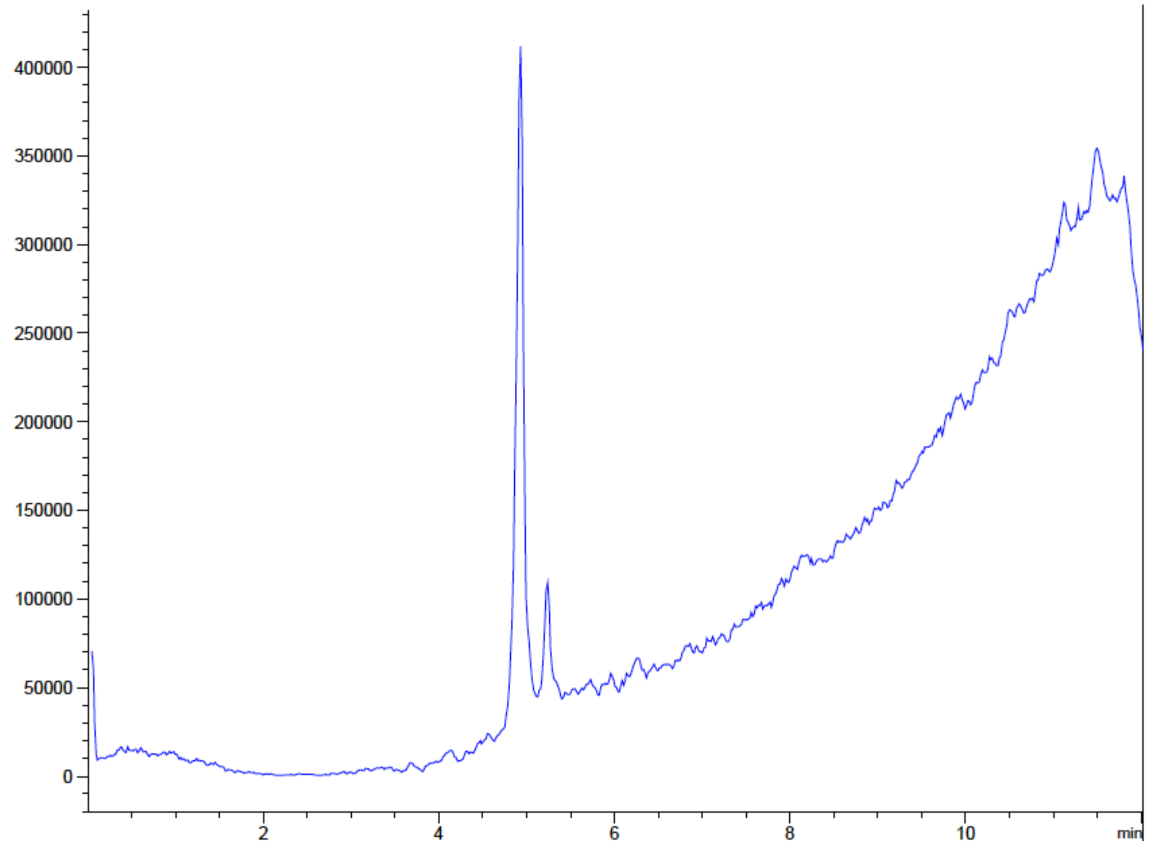

High performance liquid chromatogram of peptide 1B

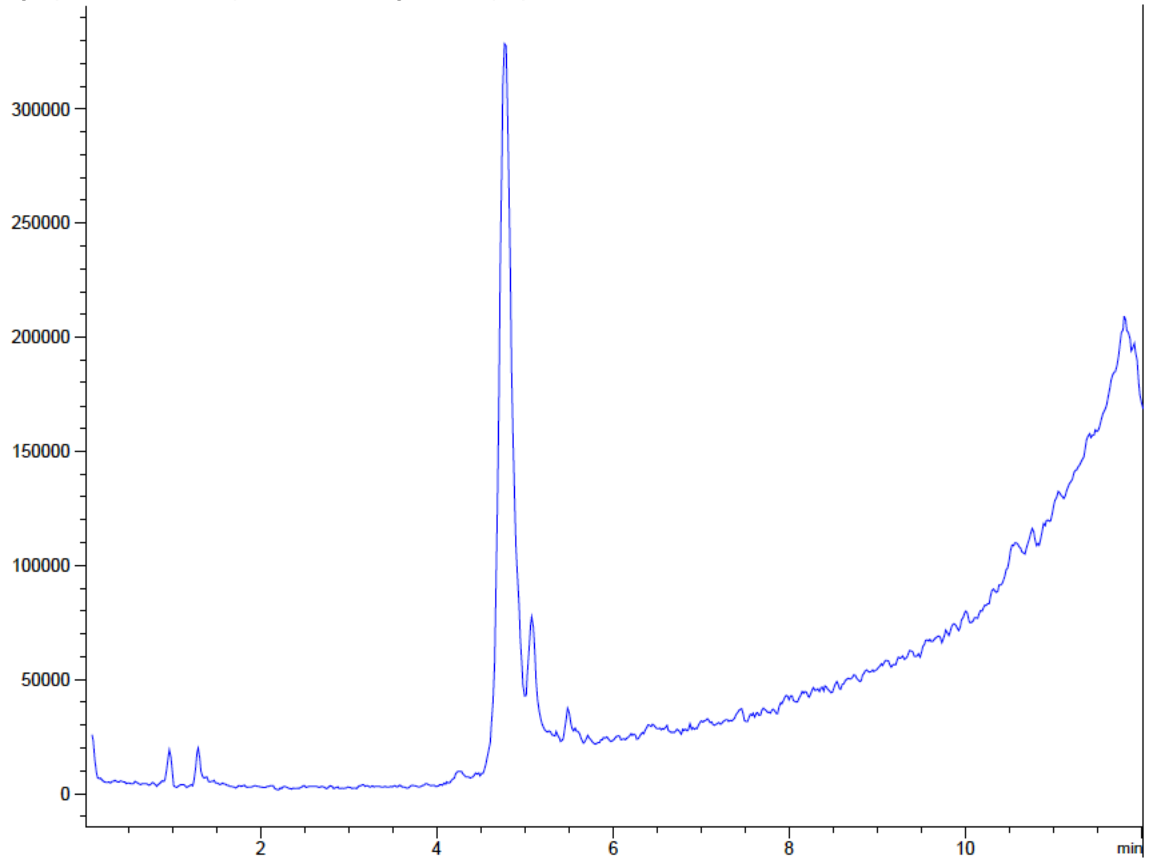

High performance liquid chromatogram of peptide 1C.

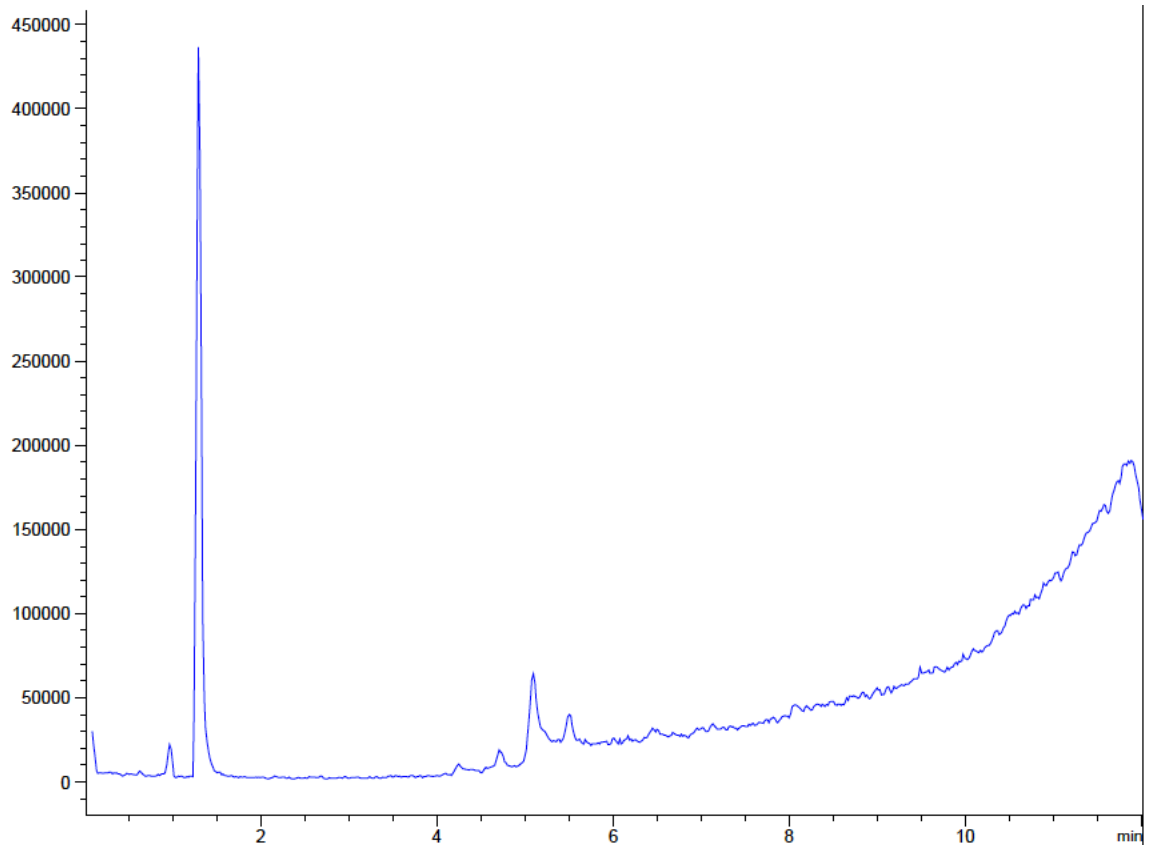

High performance liquid chromatogram of peptide 2B.

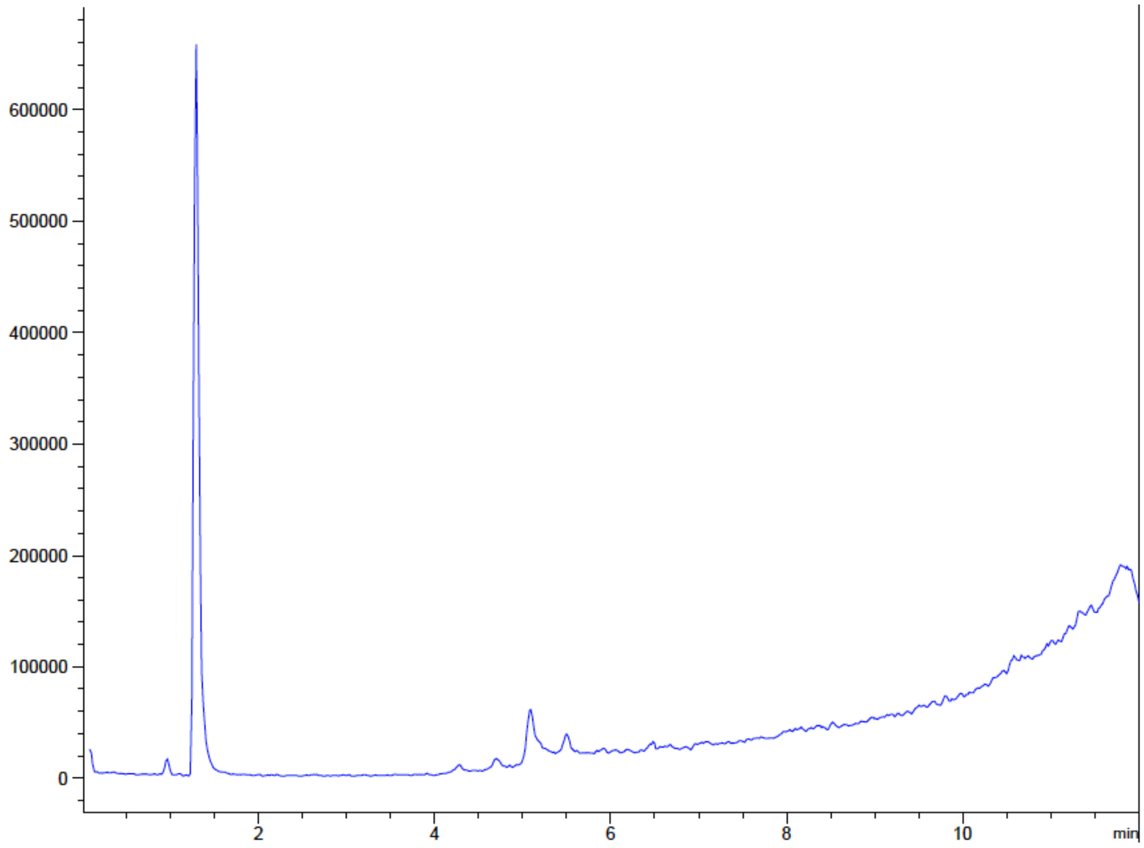

High performance liquid chromatogram of peptide 2C.

7. High performance liquid chromatography data of new peptides

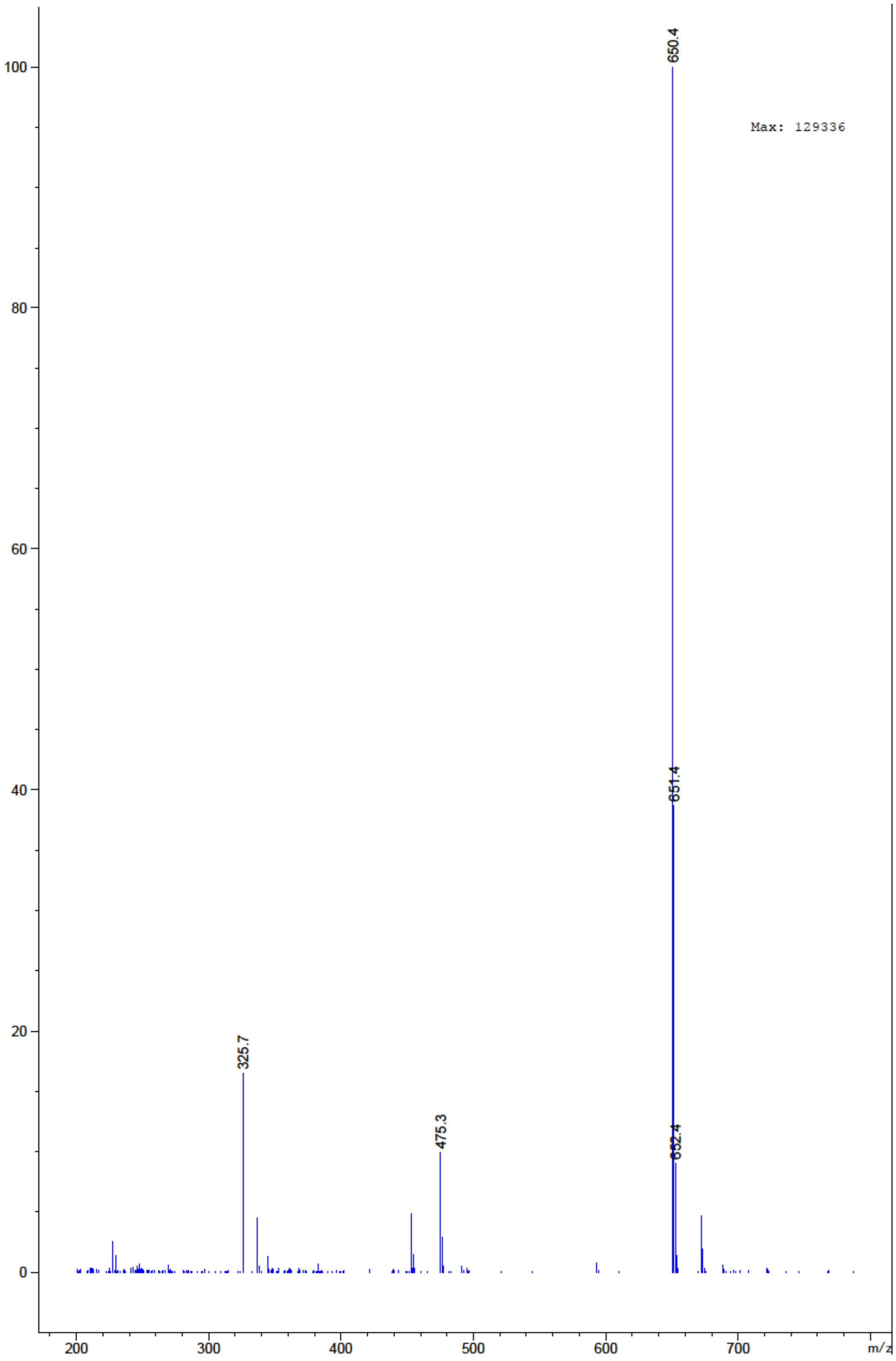

Electrospray ionisation mass spectrum of peptide 1B.
Calctd for $C_{30}H_{51}N_{9}O_{7}$, 649.79; found, 650.4 ([M + H]$^{+}$), 325.7 ([M + 2H]$^{2+}$).

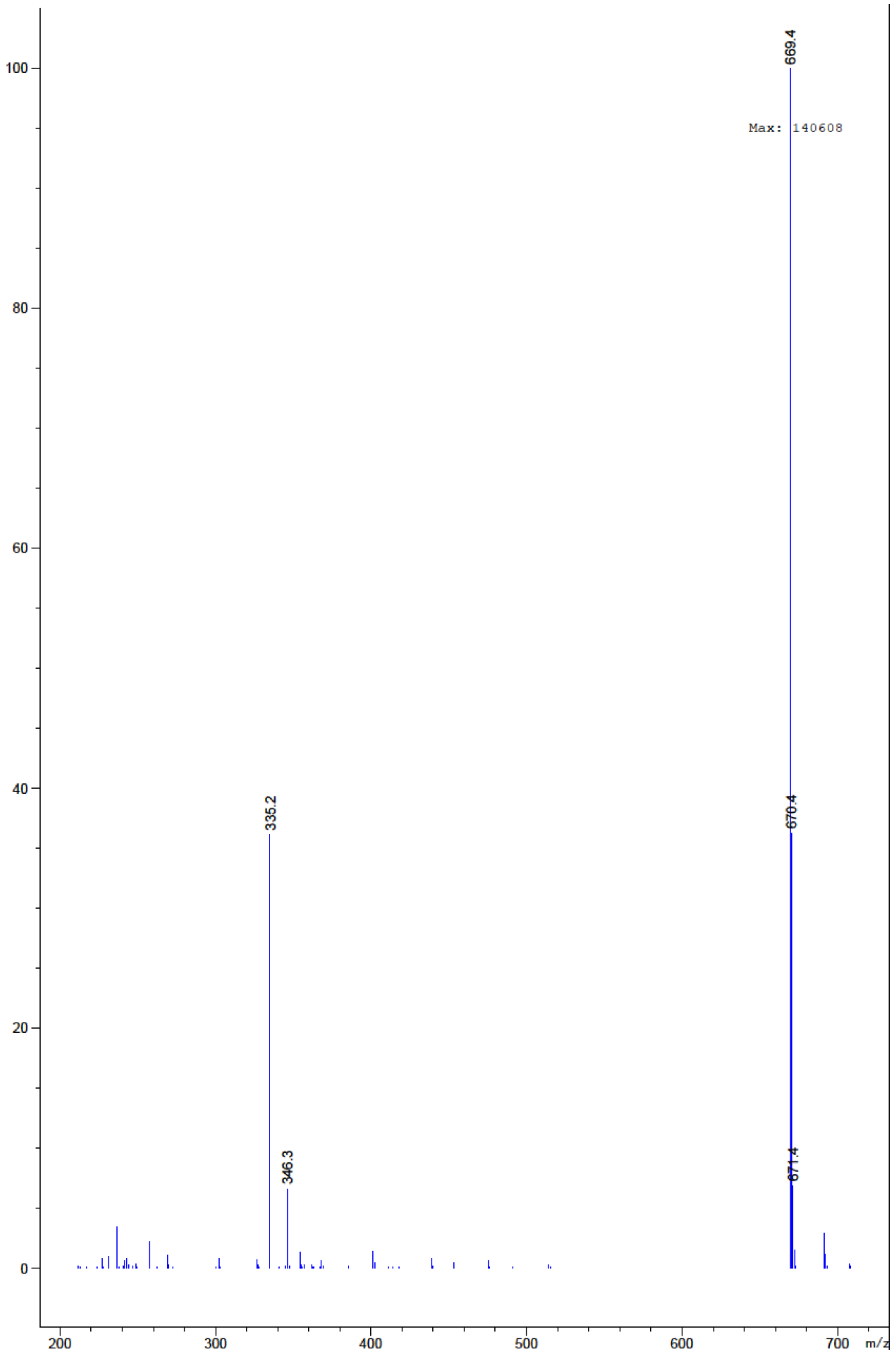

Electrospray ionisation mass spectrum of peptide 1C.
Calctd for $C_{30}H_{56}N_{10}O_7$, 668.83; found, 669.4 ([M + H]$^+$), 335.2 ([M + 2H]$^{2+}$).

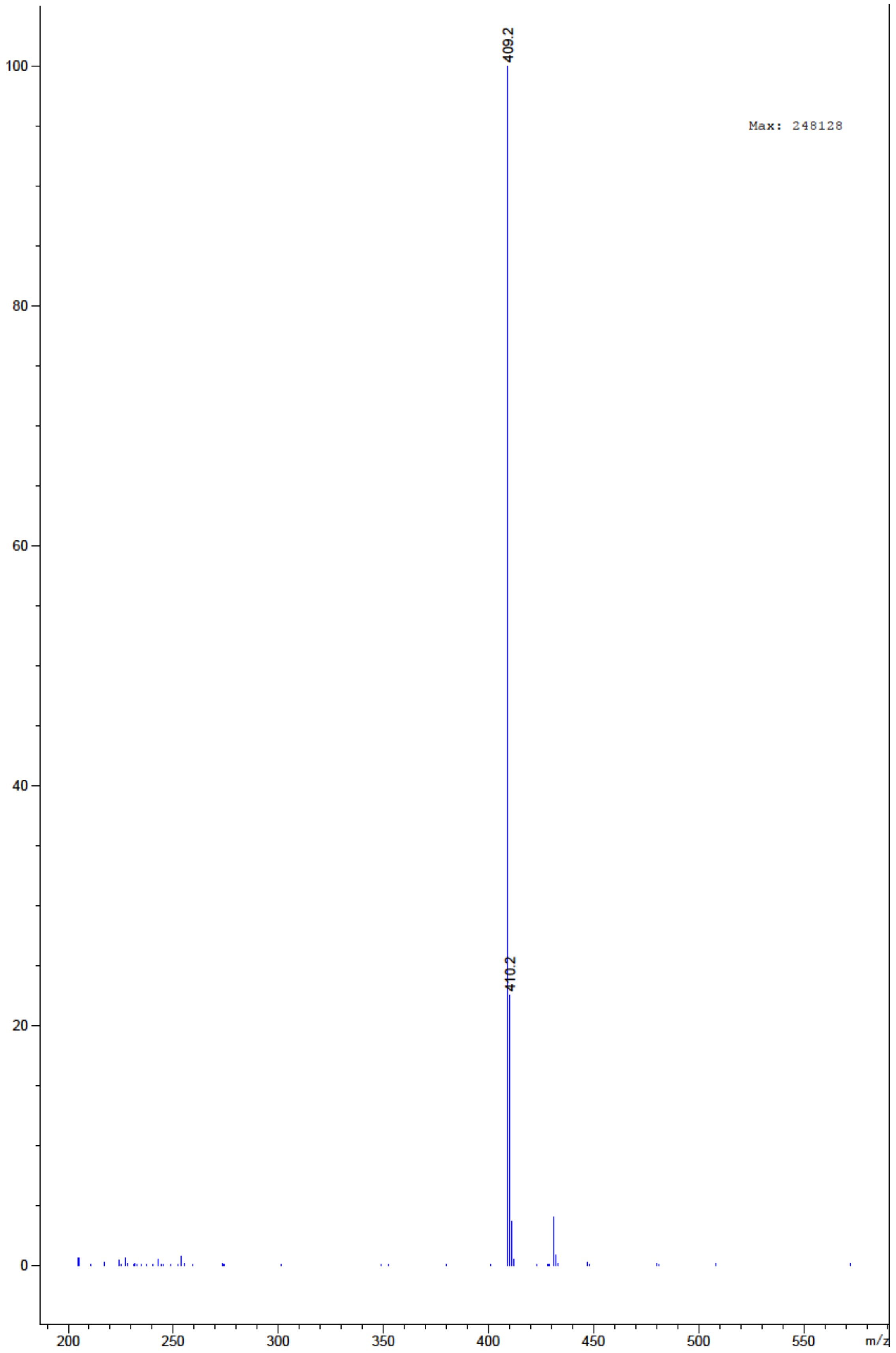

Electrospray ionisation mass spectrum of peptide 2B.
Calctd for $C_{19}H_{32}N_6O_4$, 408.50; found, 409.2 ($[M + H]^+$).

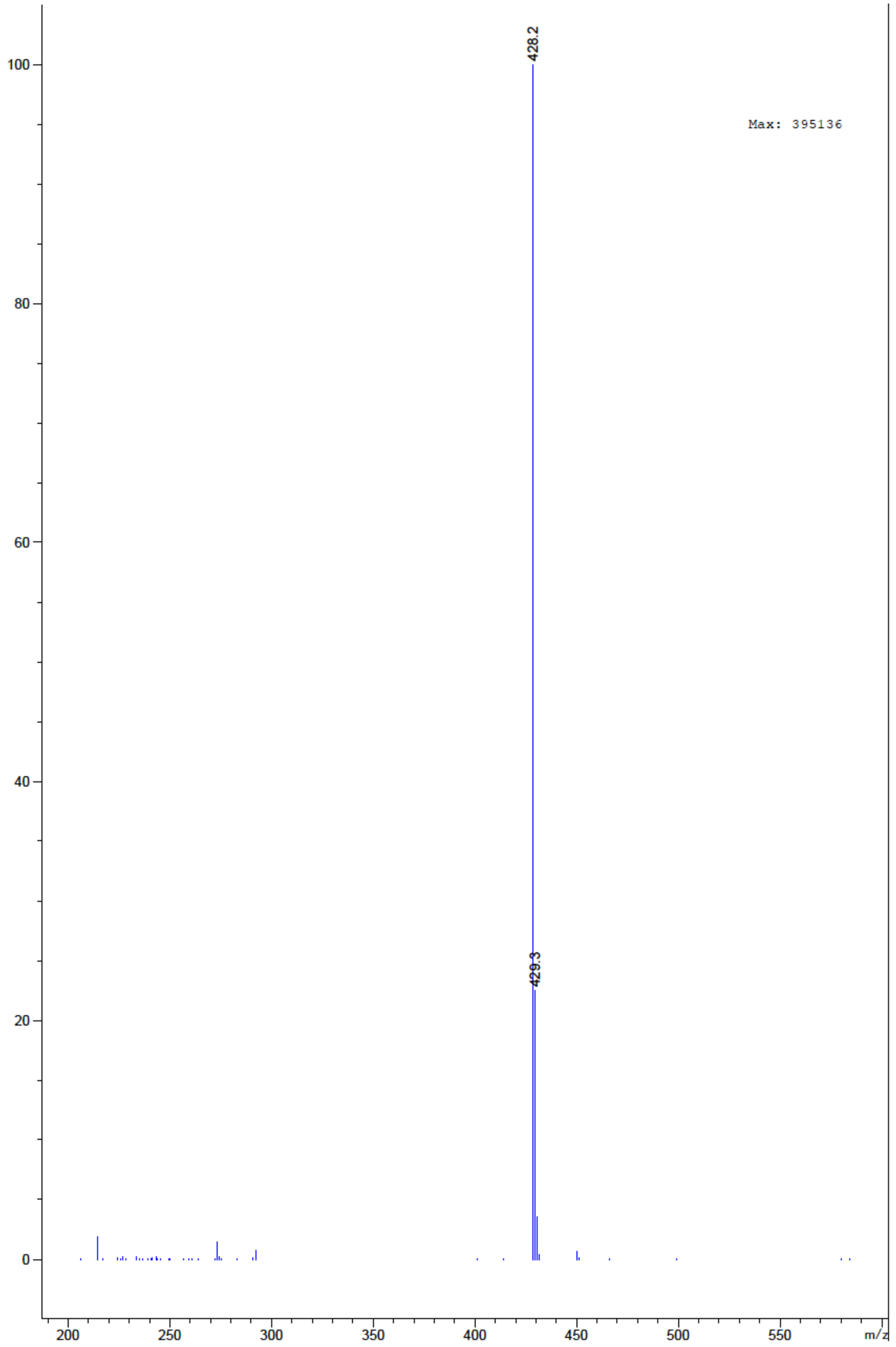

Electrospray ionisation mass spectrum of peptide 2C.
Calctd for $C_{19}H_{37}N_7O_4$, 427.54; found, 428.2 ([M + H]$^+$).
